# Combined Pectoral Nerve Block and Intravenous Dexmedetomidine in Contemporary Multimodal Breast Cancer Analgesia: A Real-World Cohort Study

**DOI:** 10.7759/cureus.116763

**Published:** 2026-09-23

**Authors:** Cornelia Weidinger, Aicha Idrissi, Khaled Abuamra, Nicole Daoud, Prasad Kulkarni, Mohamed Hassan, Vladislav Smirnov

**Affiliations:** 1 Department of Anesthesia, Dubai Hospital, Dubai Health, Dubai, ARE; 2 Department of Anesthesiology, University Hospital Erlangen, Friedrich-Alexander-Universität Erlangen-Nürnberg (FAU), Erlangen, DEU

**Keywords:** breast cancer, breast cancer post-surgery, intravenous dexmedetomidine, multimodal analgesia efficacy, opioid-sparing analgesia, pectoral nerve block, pectoral nerve block (pecs), postoperative pain

## Abstract

Background

Ultrasound-guided pectoral nerve (PECS) block and intravenous dexmedetomidine are established opioid-sparing strategies for breast cancer surgery, but in contemporary multimodal analgesic pathways, postoperative pain scores are frequently low across treatment groups, potentially limiting their ability to discriminate between analgesic approaches. This study evaluated whether complementary outcome measures provide additional insight into perioperative analgesic efficacy when combining PECS block and dexmedetomidine.

Methods

This retrospective, real-world, single-center cohort study included 292 adult females undergoing oncologic breast surgery between January 2020 and December 2024, receiving one of four routinely applied analgesic strategies: combined PECS block plus dexmedetomidine (n = 169), dexmedetomidine alone (n = 53), PECS block alone (n = 52), or neither (n = 18). The primary outcome was time to first postoperative rescue opioid administration; secondary outcomes included pain intensity, perioperative opioid consumption, heart-rate response to surgical stimulation, and safety.

Results

Postoperative pain scores remained uniformly low across all groups (median numeric rating scale score of 0 throughout the first 24 hours), and the primary outcome did not differ significantly between the combined strategy and the remaining cohort (HR: 0.83, 95% CI: 0.198-3.47; p = 0.798). Combined PECS block and dexmedetomidine was associated with lower unadjusted intraoperative opioid consumption (17.7 vs. 50.7 morphine milligram equivalents; p < 0.001). However, this difference tracked almost entirely with intraoperative remifentanil use rather than with analgesic strategy, and largely disappeared once remifentanil-exposed cases were excluded. Heart-rate attenuation between induction and incision tracked with receipt of a PECS block rather than with dexmedetomidine. Rescue opioid use, pain scores, and clinically relevant bradycardia were comparable between groups throughout.

Conclusions

Postoperative pain scores and opioid requirements were uniformly low regardless of analgesic strategy, and the primary time-to-rescue-opioid outcome was inconclusive. Both apparent secondary "positive" findings, the opioid-sparing effect and the heart-rate attenuation, were attributable to confounding by anesthetic maintenance technique and PECS block status, respectively, rather than to the combined strategy itself. These findings illustrate a floor effect in postoperative pain scores under contemporary multimodal pathways, and highlight anesthetic-technique confounding, calendar-time drift, and single-clinician decision-making as underappreciated sources of bias in retrospective analgesia research more broadly.

## Introduction

Over the past two decades, multimodal perioperative analgesia has transformed postoperative pain management after breast cancer surgery. While this progress has substantially improved patient outcomes, it has also created a new challenge for clinical research: distinguishing meaningful differences between increasingly effective analgesic strategies. The routine integration of regional anesthesia techniques, non-opioid analgesics, and opioid-sparing anesthetic strategies has substantially improved early recovery while reducing opioid exposure and postoperative morbidity [1-5]. Nevertheless, breast cancer surgery remains associated with a considerable risk of persistent postsurgical pain (PPSP), and inadequate control of acute postoperative pain continues to be recognized as one of its most important modifiable risk factors [6-11]. Optimizing perioperative analgesia therefore remains a major clinical objective despite the considerable progress achieved over recent decades.

Among regional anesthesia techniques, ultrasound-guided pectoral nerve (PECS) blocks have become an established component of multimodal analgesia for breast surgery because they provide effective analgesia of the anterolateral chest wall while reducing perioperative opioid requirements [12-15]. Similarly, intravenous dexmedetomidine has gained increasing acceptance as an adjunct to general anesthesia because of its analgesic, opioid-sparing, sympatholytic, and sedative properties [16-18]. In addition to its systemic effects, dexmedetomidine has been shown to prolong regional analgesia when administered as a perineural adjunct [19-21] and may contribute to improved longer-term pain outcomes following breast cancer surgery [22]. Consequently, both interventions are widely incorporated into contemporary opioid-sparing perioperative pathways.

Although the individual benefits of PECS block and dexmedetomidine have been demonstrated in numerous randomized trials and meta-analyses, considerably less evidence is available regarding their combined use within established multimodal analgesic pathways. Most previous studies compared a single intervention with conventional analgesic management, whereas comparatively few have evaluated whether combining regional and systemic opioid-sparing strategies provides additional benefit once high-quality multimodal analgesia has already been implemented in routine clinical practice.

This question has become increasingly relevant because the success of contemporary multimodal analgesia has introduced a methodological challenge for perioperative research. As postoperative pain scores approach the lower limit of conventional numerical rating scales, their ability to discriminate between effective analgesic strategies diminishes, creating a floor effect that may obscure clinically meaningful differences [3,4,23-26]. Under these circumstances, complementary outcome measures, including perioperative opioid exposure, autonomic responses to surgical stimulation, and recovery-related parameters, may provide a more sensitive assessment of analgesic performance than pain scores alone.

The present study therefore evaluated the combined use of ultrasound-guided PECS block and intravenous dexmedetomidine in patients undergoing breast cancer surgery within a contemporary real-world multimodal analgesic pathway. The primary aim was to determine whether the combined PECS block and dexmedetomidine strategy differed from other routinely applied strategies with respect to time to first postoperative rescue opioid administration, defined a priori as the single primary outcome; postoperative pain intensity, perioperative opioid consumption, and heart-rate response to surgical stimulation were examined as secondary and exploratory outcomes, on the hypothesis, rather than the premise, that they might provide complementary information beyond postoperative pain scores alone.

## Materials and methods

Study design and ethical approval

This retrospective observational cohort study was conducted at a tertiary referral center specializing in breast cancer surgery in Dubai, United Arab Emirates, between January 2020 and December 2024. Cases from calendar year 2019 were not included in data collection because of extensive missing or inconsistent perioperative documentation associated with incomplete implementation of the institutional EPIC® system (Epic Systems Corporation, Verona, WI) during that year; a precise count of 2019 cases considered and excluded was not retained.

Perioperative data were extracted from the institutional electronic health record system (EPIC®). Ethical approval was obtained from the Dubai Scientific Research Ethics Committee (DSREC-07/2025_11) and the Mohammed Bin Rashid University Institutional Review Board (MBRU IRB-2025-418). Owing to the retrospective nature of the study and the use of routinely collected, de-identified clinical data, the requirement for individual informed consent was waived. The study was reported in accordance with the STROBE (Strengthening the Reporting of Observational Studies in Epidemiology) guidelines for observational research [27].

Study population

The study population comprised consecutive adult females (≥18 years) undergoing oncologic breast surgery (mastectomy or lumpectomy, with or without axillary procedures) under general anesthesia. Inclusion required complete perioperative documentation and the availability of numeric rating scale (NRS) pain assessments. Exclusion criteria comprised non-oncologic procedures, revision or day-case surgery, age below 18 years, and missing essential perioperative data. A total of 292 patients met the eligibility criteria and were included in the final analysis. Pre-inclusion screening counts were not preserved as a separate log during the retrospective data collection process; this is acknowledged as a limitation on the completeness of the reported cohort-assembly process.

Sample size and analytical strategy

No formal a priori power calculation was performed. The study cohort represents all consecutive eligible cases during the defined study period and was intended to characterize real-world clinical outcomes rather than to provide confirmatory hypothesis testing. As all eligible patients during the study period were included, the cohort reflects routine clinical practice without additional sampling. Accordingly, the sample size allows descriptive analysis and exploration of between-group trends but is not powered to detect small effect sizes. Subgroup analyses according to surgical extent (mastectomy vs. lumpectomy) and analgesic strategy were predefined and considered exploratory.

Perioperative analgesic strategies

Patients received one of four routinely applied perioperative analgesic strategies according to clinical judgement and the attending anesthesiologist's routine practice.

These analgesic strategies were either the combination of PECS block combined with intravenous dexmedetomidine, intravenous dexmedetomidine only, PECS block only, or neither PECS block nor dexmedetomidine. Patients in the latter group received the institutional multimodal baseline regimen (scheduled paracetamol ± nonsteroidal anti-inflammatory drug, standard general anesthesia, and intraoperative opioid titration at clinician discretion) but neither of the two studied adjuncts; the term "neither" refers only to the absence of these two specific interventions, not to the absence of intraoperative analgesia.

The primary comparison contrasted the combined PECS block and intravenous dexmedetomidine strategy with all other approaches pooled together, addressing whether the combined strategy adds benefit over routine practice not combining both interventions. A complementary four-group analysis was performed to address the distinct question of whether the components have additive or synergistic effects individually.

After administration of general anesthesia and multimodal analgesia, the administration of adjuncts followed institutional standards. Dexmedetomidine dosing and PECS block techniques, as well as administration of local anesthesia, are described below.

Dexmedetomidine was administered intravenously with a loading dose of 0.6-1.0 μg/kg over 15 minutes in all patients allocated to the dexmedetomidine group. In selected cases, this was followed by a continuous infusion of 0.2-0.4 μg/kg/h, titrated according to intraoperative hemodynamic response and anticipated surgical duration at the discretion of the attending anesthesiologist. No predefined hemodynamic thresholds governed the decision to initiate a continuous infusion.

PECS blocks were performed as ultrasound-guided PECS II blocks without catheter placement. The PECS block was performed under ultrasound guidance using ropivacaine 0.2-0.375%, with a total volume of 30-40 mL depending on patient characteristics and surgical extent. As routine practice, approximately 10 mL was injected between the pectoralis major and minor (PECS I component), with the remaining volume injected between the pectoralis minor and serratus anterior (PECS II extension); the exact per-patient volume split was not individually documented or analyzed.

All data regarding anesthesia and multimodal analgesia administration were extracted from the electronic anesthesia record.

Perioperative monitoring and pain assessment

Standard intraoperative monitoring included electrocardiography, non-invasive blood pressure measurement, pulse oximetry, and capnography; invasive arterial monitoring was used when clinically indicated. Heart rate recorded at induction of anesthesia and at surgical incision was used as a surrogate marker of autonomic and nociceptive response.

Postoperative pain was assessed using the NRS (0-10) in the post-anesthesia care unit (PACU) and at four, six, 12, and 24 hours after surgery. Rest pain and dynamic pain were not separately captured in the institutional documentation, as the rescue medication was given only on request. Maximum NRS values in the PACU and during the first 24 postoperative hours were analyzed.

Postoperative analgesic regimen

Postoperative analgesia followed an institutional multimodal postoperative analgesic protocol consisting of scheduled paracetamol with or without nonsteroidal anti-inflammatory drugs, unless contraindicated. Additionally, on patient request, rescue analgesia includes tramadol, nalbuphine, or intramuscular morphine as clinically indicated. Following the opioid-sparing strategy of our institution, opioids are not administered in a scheduled pathway. All intraoperative and postoperative opioid doses were converted to intravenous morphine milligram equivalents (MME) using standardized institutional conversion factors (Table 1).

**Table 1:** Conversion factors for intravenously administered opioids expressed as intravenous morphine milligram equivalents (i.v. MME). Opioid equivalence factors were derived from the University of California, San Francisco oral morphine equivalent framework [28] and normalized to intravenous morphine. As 1 mg intravenous morphine corresponds to 3 mg oral morphine equivalents in this framework, oral morphine equivalent values for intravenously administered opioids were divided by three to express doses relative to 1 mg intravenous morphine. Abbreviations: i.v. MME = intravenous morphine milligram equivalents; OME = oral morphine equivalents; i.v. = intravenous; mg = milligrams; mcg = micrograms; UCSF = University of California, San Francisco.

Morphine milligram equivalents (MME)				
Opioid (i.v.)	Dosage	Morphine (i.v.)	Conversion factor	Reference
Morphine	1 mg	1 mg	1	UCSF [28]
Fentanyl	100 mcg	10 mg	1 mcg = 0.1 i.v. MME	UCSF [28]
Remifentanil	100 mcg	10 mg	1 mcg = 0.1 i.v. MME	UCSF [28]
Oxycodone	1 mg	1.5 mg	1 mg = 1.5 i.v. MME	UCSF [28]
Nalbuphine	1 mg	1 mg	1 mg = 1 i.v. MME	UCSF [28]
Tramadol	10 mg	1 mg	1 mg = 0.1 i.v. MME	UCSF [28]

Outcome measures

Because postoperative pain scores were expected to be low within the institutional multimodal analgesic pathway and all patients received standardized scheduled non-opioid analgesia, outcomes were selected to evaluate complementary dimensions of perioperative analgesic efficacy beyond pain intensity alone. The predefined outcome measures therefore captured three complementary domains of perioperative analgesic performance: patient-centered outcomes, physiological response to surgical stimulation, and safety outcomes.

Primary outcome

The primary outcome was time to first postoperative rescue opioid administration, defined as the interval between admission to the PACU and documented administration of the first rescue opioid within the first 24 postoperative hours. For this outcome, only opioids administered in the surgical ward were counted as a rescue-opioid event. Opioids administered in the PACU were not counted as a primary-outcome event, since PACU care remains under direct intraoperative anesthesiology management rather than representing a ward-level escalation of the postoperative analgesic protocol. PACU opioid administration is reported separately as a safety measure. Rescue opioids were administered on an as-needed basis according to institutional practice and clinical/patient-reported need, without a mandated fixed NRS threshold. Patients who did not receive a ward rescue opioid during the observation period were censored at 24 hours for time-to-event analyses.

Secondary outcomes

The secondary outcomes were postoperative analgesic effectiveness and physiological outcomes. The postoperative analgesic effectiveness was measured as patient-centered outcomes, including postoperative pain intensity (maximum NRS score in the PACU and maximum NRS during the first 24 postoperative hours), postoperative opioid consumption expressed as intravenous MME, cumulative non-opioid analgesic consumption, and the proportion of patients requiring postoperative rescue opioids. The physiological outcome comprised intraoperative opioid consumption, heart rate response to surgical stimulation, and clinically relevant bradycardia requiring anticholinergic treatment.

Exploratory analyses

The prespecified exploratory analyses included procedure-specific comparisons between mastectomy and lumpectomy and comparisons across the four routinely applied perioperative analgesic strategies to explore potential additive or synergistic effects of PECS block and intravenous dexmedetomidine.

Statistical analysis

Continuous variables were analyzed using Welch’s t-test or the Brunner-Munzel test, as appropriate. Categorical variables were compared using Fisher’s exact test. Time-to-event outcomes were evaluated using Kaplan-Meier analysis with log-rank testing and Cox proportional hazards regression. All tests were two-sided, with a significance threshold of p < 0.05. Given the non-randomized design, analyses were considered descriptive and hypothesis-generating. No adjustment for multiple testing was performed because secondary and subgroup analyses were considered exploratory. A data analysis and statistical plan was written and filed with the institutional review board before data were accessed.

Sensitivity analyses were conducted to assess the robustness of the primary findings. These included procedure-restricted analyses (mastectomy-only and lumpectomy-only cohorts), alternative comparator definitions (four-group analyses), alternative pain and analgesic endpoints, and robust non-parametric analyses accounting for skewed distributions.

A multivariable linear regression was performed for the continuous intraoperative opioid consumption outcome, adjusting for surgical procedure (mastectomy vs. lumpectomy), to account for potential imbalance in surgical extent between analgesic-strategy groups. A further sensitivity analysis restricted this regression to patients who did not receive intraoperative remifentanil, given the possibility that anesthetic maintenance technique differs between groups. The proportion of patients receiving combined PECS block and dexmedetomidine by calendar year was compared using the chi-square test to assess potential temporal confounding. The proportional hazards assumption for the primary Cox model was assessed using Schoenfeld residuals. Given the small number of observed primary-outcome events, no additional covariates were added to the primary Cox model, in accordance with the general guideline of at least 10 events per model covariate. Missing perioperative data were not imputed. Patients with missing essential perioperative data were excluded at the point of data collection.

Reporting

The study is reported in accordance with the STROBE statement [27]. The reporting framework was applied to the study design, cohort, outcomes, statistical methods, and interpretation of the observational findings.

## Results

Study population

A total of 292 consecutive patients met the predefined eligibility criteria and were included in the final analysis. Of these, 169 (57.9%) patients received combined ultrasound-guided PECS block and intravenous dexmedetomidine, whereas 123 (42.1%) patients received alternative multimodal analgesic strategies, including dexmedetomidine alone, PECS block alone, or neither intervention (Figure 1).

**Figure 1:**
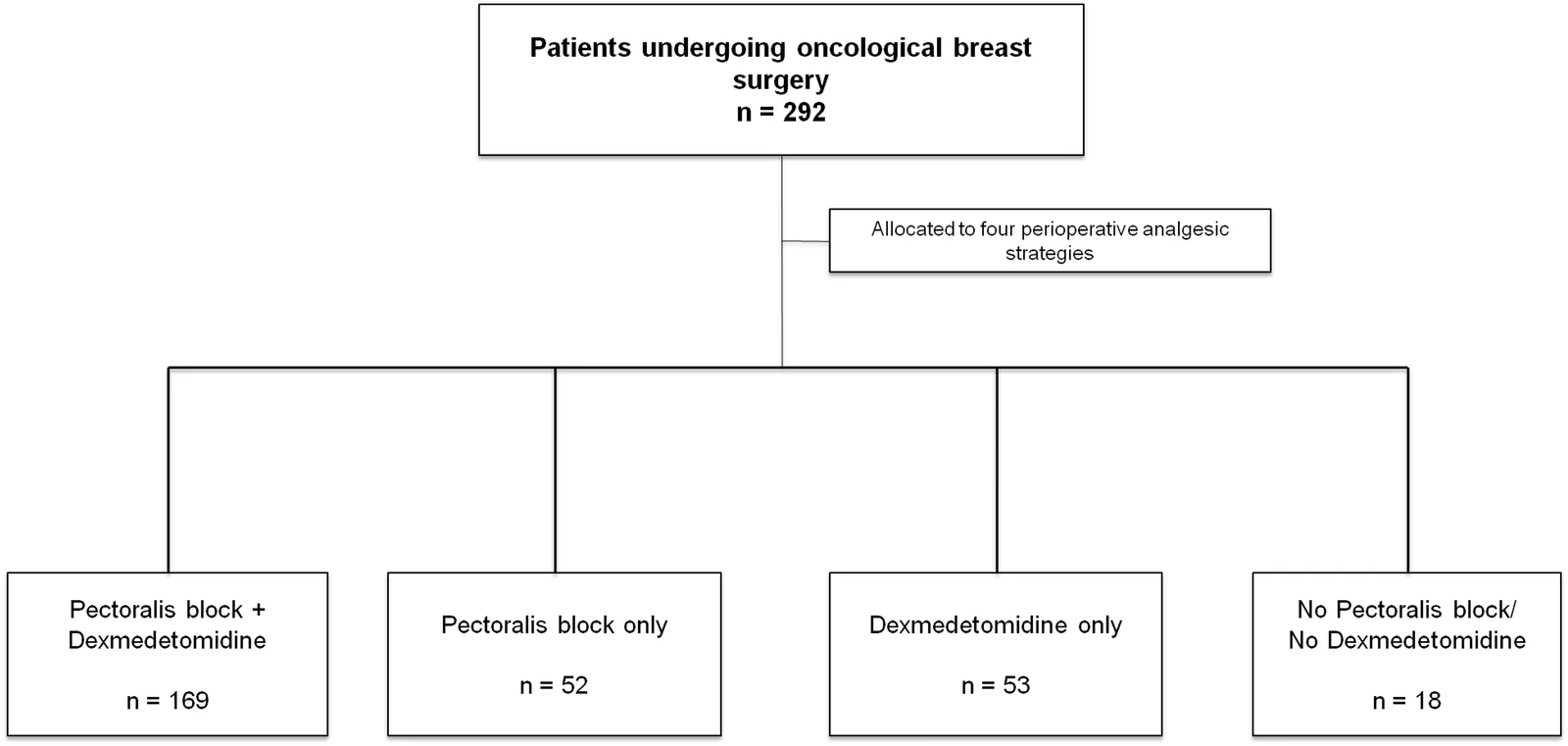
Study flow and allocation of included patients. Flow of included patients (n = 292) and allocation to four perioperative analgesic strategies: pectoral block + dexmedetomidine; dexmedetomidine only; pectoral block only; and no pectoral block/no dexmedetomidine. Abbreviations: n = sample size.

Baseline characteristics

Baseline demographic characteristics (age, weight, BMI, American Society of Anesthesiologists (ASA) status) were comparable between groups. Surgical procedure, however, differed significantly, with mastectomy performed more frequently in the combined PECS + dexmedetomidine group (68.6% vs. 39.8%; p < 0.001) and lumpectomy more frequent in the comparator group (31.4% vs. 60.2%). Because surgical extent plausibly influences intraoperative opioid requirement, this imbalance is addressed via the procedure-adjusted analysis reported below.

The mean age was comparable (50.7 ± 11.3 vs. 52.5 ± 12.8 years), as were body weight (70.0 ± 16.6 kg vs. 71.9 ± 15.3 kg), BMI (28.6 ± 6.3 vs. 29.2 ± 6.0 kg/m²), and ASA physical status.

The study cohort reflected routine clinical practice, with breast-conserving surgery representing the predominant procedure in both treatment groups, although patients receiving the combined PECS block and dexmedetomidine strategy underwent mastectomy more frequently than patients treated with alternative analgesic strategies (116 (68.6%) versus 49 (39.8%)), whereas lumpectomy was performed more often in the comparator group (74 (60.2%) versus 53 (31.4%)). Rates of axillary surgery, including sentinel lymph node biopsy and axillary lymph node dissection, were comparable between groups. Procedure-specific baseline characteristics for the combined cohort (mastectomy and lumpectomy) are presented in Table 2 and stratified by surgical procedure in Table 3.

**Table 2:** Baseline characteristics (mastectomy + lumpectomy). Baseline demographic and surgical characteristics in pectoral nerve block plus dexmedetomidine versus other strategies. Continuous data are presented as mean (SD) and median (IQR); categorical data as n (%). Continuous variables were compared using Welch’s t-test or the Brunner–Munzel test, as appropriate, and categorical variables using Fisher’s exact test. P < 0.05 was considered statistically significant. Abbreviations: SD = standard deviation; IQR = interquartile range; BMI = body mass index; ASA = American Society of Anesthesiologists; kg = kilograms; m = meters; n = sample size.

Characteristics		Pectoral block + dexmedetomidine	Other analgesic strategies	P
		n = 169	n = 123	
Age (years)	Mean (SD)	50.7 (11.3)	52.5 (12.8)	0.328
	Median (Q1; Q3)	49 (43; 58)	51 (42; 61)	
Body weight (kg)	Mean (SD)	70 (16.6)	71.9 (15.3)	0.233
	Median (Q1; Q3)	68 (59; 78.2)	69.5 (60.9; 79.9)	
BMI (kg/m²)	Mean (SD)	28.6 (6.3)	29.2 (6)	0.306
	Median (Q1; Q3)	27.4 (24.6; 31.2)	28 (24.6; 32.3)	
ASA classification	1	12/169 (7.1%)	14/123 (11.4%)	0.812
	2	97/169 (57.4%)	63/123 (51.2%)	
	3	58/169 (34.3%)	46/123 (37.4%)	
	4	2/169 (1.2%)	0/123 (0%)	
Surgical procedure	Mastectomy	116/169 (68.6%)	49/123 (39.8%)	<0.001
	Lumpectomy	53/169 (31.4%)	74/123 (60.2%)	
Lymph node surgery		18/169 (10.7%)	11/123 (8.9%)	0.642

**Table 3:** Baseline characteristics stratified by surgical procedure. Baseline demographic and surgical characteristics in pectoral nerve block plus dexmedetomidine versus other strategies. Continuous data are presented as mean (SD) and median (IQR); categorical data as n (%). Continuous variables were compared using Welch’s t-test or the Brunner–Munzel test, as appropriate, and categorical variables using Fisher’s exact test. P < 0.05 was considered statistically significant. Abbreviations: SD = standard deviation; IQR = interquartile range; BMI = body mass index; ASA = American Society of Anesthesiologists; kg = kilograms; m = meters; n = sample size.

Mastectomy only (n = 165)					Lumpectomy only (n = 127)				
Characteristics		Pectoral block + dexmedetomidine	Other analgesic strategies	P	Characteristics		Pectoral block + dexmedetomidine	Other analgesic strategies	P
		n = 116	n = 49				n = 53	n = 74	
Age (years)	Mean (SD)	50.2 (10.7)	51.4 (14.6)	0.911	Age (years)	Mean (SD)	51.6 (12.6)	53.3 (11.6)	0.513
	Median (Q1; Q3)	48 (42.8; 56.2)	48 (41; 61)			Median (Q1; Q3)	50 (43; 62)	52.5 (43.2; 61)	
Body weight (kg)	Mean (SD)	68.4 (17.6)	65.8 (14.1)	0.435	Body weight (kg)	Mean (SD)	73.6 (13.7)	75.9 (14.8)	0.514
	Median (Q1; Q3)	65 (56.5; 76.2)	63.9 (55; 73.5)			Median (Q1; Q3)	72 (63; 81.7)	75 (64; 82)	
BMI (kg/m²)	Mean (SD)	28 (6.8)	26.8 (5.8)	0.272	BMI (kg/m²)	Mean (SD)	29.8 (4.9)	30.8 (5.7)	0.511
	Median (Q1; Q3)	26.6 (23.6; 30.8)	25.3 (22.9; 29.5)			Median (Q1; Q3)	30 (26.6; 33.1)	29.8 (26.9; 33.6)	
ASA classification	1	9/116 (7.8%)	8/49 (16.3%)	0.164	ASA classification	1	3/53 (5.7%)	6/74 (8.1%)	0.204
	2	61/116 (52.6%)	25/49 (51%)			2	36/53 (67.9%)	38/74 (51.4%)	
	3	44/116 (37.9%)	16/49 (32.7%)			3	14/53 (26.4%)	30/74 (40.5%)	
	4	2/116 (1.7%)	0/49 (0%)			4	0/53 (0%)	0/74 (0%)	
Lymph node surgery		14/116 (12.1%)	4/49 (8.2%)	0.488	Lymph node surgery		4/53 (7.5%)	7/74 (9.5%)	0.729

Anesthetic management was largely consistent across the study cohort. The proportions of patients receiving total intravenous anesthesia and volatile anesthesia were similar. As expected, dexmedetomidine exposure differed according to the allocated analgesic strategy, while all other aspects of perioperative care followed the institutional multimodal analgesic protocol.

Primary outcome: Time to first opioid administration

The time to first postoperative rescue opioid administration, defined as the first ward-administered rescue opioid, was comparable between patients receiving combined PECS block and intravenous dexmedetomidine and those treated with alternative multimodal analgesic strategies (Figure 2 and Table 4). Cox proportional hazards analysis showed no statistically significant difference between groups (hazard ratio (HR): 0.83, 95% CI: 0.198-3.47; p = 0.798), consistent with the log-rank test (p = 0.798). The proportional hazards assumption was not violated (Schoenfeld residuals, p = 0.14), although this check is likely underpowered given the small number of events. Only eight patients across the entire cohort received a ward rescue opioid within the study period. The 95% CI accordingly spans both a substantial reduction and a substantial increase in hazard.

**Figure 2:**
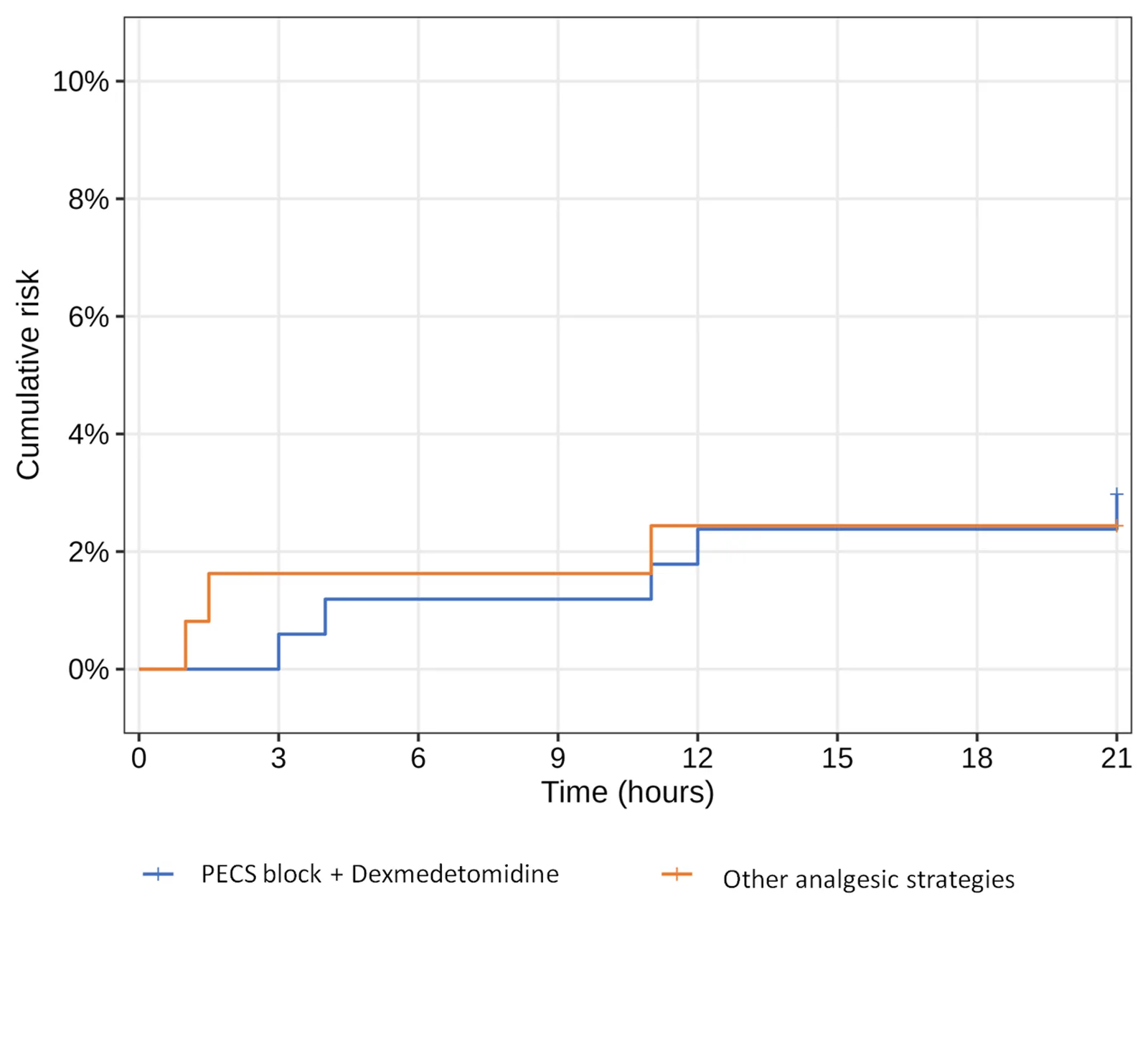
Time to first postoperative rescue opioid administration (mastectomy and lumpectomy). Kaplan–Meier curves compare the combined pectoral nerve block plus dexmedetomidine strategy with other analgesic strategies. Abbreviations: PECS = pectoral nerve block.

**Table 4:** Time to first opioid administration (mastectomy + lumpectomy). Time-to-event outcomes were evaluated using Kaplan–Meier analysis with log-rank testing and Cox proportional hazards regression. P < 0.05 was considered statistically significant. Abbreviations: PECS = pectoral nerve block; HR = hazard ratio; CI = confidence interval.

Time (hours)	PECS block + dexmedetomidine		Other analgesic strategies		
3	0.6 (0; 1.8)		1.6 (0; 3.8)		
6	1.2 (0; 2.8)		1.6 (0; 3.8)		
12	2.4 (0; 4.7)		2.4 (0; 5.1)		
20	2.4 (0; 4.7)		2.4 (0; 5.1)		
Comparison		HR		95% CI	P
Other analgesic strategies		0.83		0.198; 3.47	0.798

The Kaplan-Meier curves for the two groups were closely similar throughout the observation period (Figure 2), consistent with the small number of observed events.

Estimated time-to-opioid administration showed a similar pattern in both groups across the first 24 postoperative hours, consistent with the findings of the Kaplan-Meier analysis.

Prespecified exploratory subgroup analyses according to surgical procedure are presented in Figure 3 and Table 5. None of these exploratory subgroup comparisons reached statistical significance and should therefore be interpreted as hypothesis-generating.

**Figure 3:**
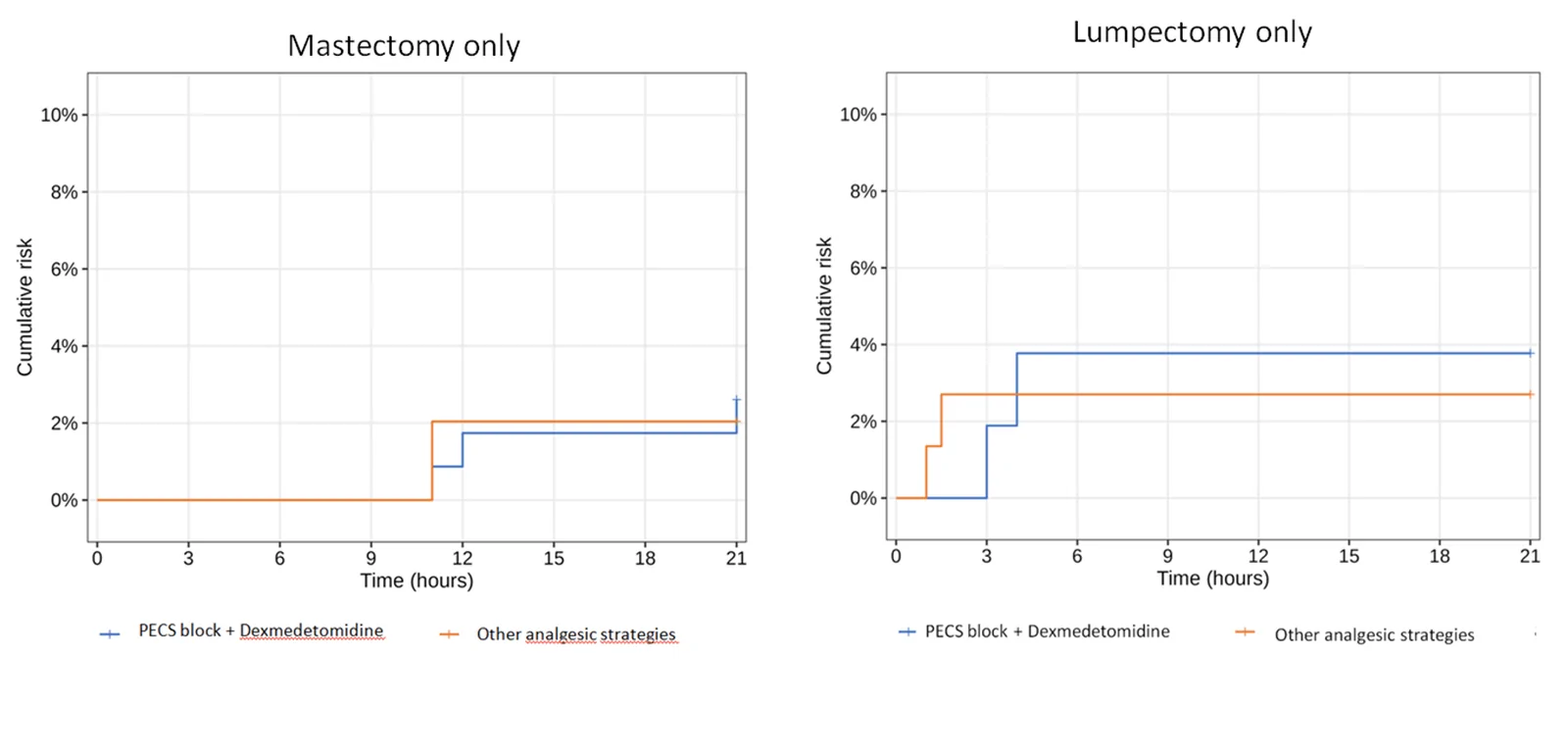
Procedure-specific time to first postoperative rescue opioid administration stratified by surgical procedure. Cumulative risk is shown over postoperative time for the combined pectoral nerve block plus dexmedetomidine strategy and other analgesic strategies. Abbreviations: PECS = pectoral nerve block.

**Table 5:** Procedure-specific time to first postoperative rescue opioid administration stratified by surgical procedure. Time-point values are cumulative risk estimates with 95% confidence intervals where shown. Time-to-event outcomes were evaluated using Kaplan–Meier analysis with log-rank testing and Cox proportional hazards regression. P < 0.05 was considered statistically significant. The reference group for the hazard ratio is "Other analgesic strategies"; HR < 1 favors the combined PECS + dexmedetomidine group. Abbreviations: PECS = pectoral nerve block; HR = hazard ratio; CI = confidence interval.

Mastectomy only						Lumpectomy only					
Time (hours)	Pectoral block + dexmedetomidine		Other analgesic strategies			Time (hours)	Pectoral block + dexmedetomidine		Other analgesic strategies		
3	0 (0; 0)		0 (0; 0)			3	1.9 (0; 5.5)		2.7 (0; 6.3)		
6	0 (0; 0)		0 (0; 0)			6	3.8 (0; 8.8)		2.7 (0; 6.3)		
12	1.7 (0; 4.1)		2 (0; 5.9)			12	3.8 (0; 8.8)		2.7 (0; 6.3)		
20	1.7 (0; 4.1)		2 (0; 5.9)			20	3.8 (0; 8.8)		2.7 (0; 6.3)		
Comparison		HR		95% CI	p	Comparison		HR		95% CI	p
Other analgesic strategies		0.79		0.08; 7.56	0.835	Other analgesic strategies		0.73		0.10; 5.15	0.748

Postoperative analgesic effectiveness

Pain Intensity

The overall postoperative pain burden was remarkably low throughout the first 24 hours following surgery. Median NRS pain scores remained 0 at all assessed time points in both treatment groups, including the PACU and at 4, 6, 12, and 24 hours after surgery (Figures 4, 5 and Table 6). Across the entire cohort, the proportion of patients with NRS ≥ 4 at any time point remained low: 7.5% in the PACU, and 1.4-3.8% at the 4-, 6-, 12-, and 24-hour ward assessments, with no consistent pattern favoring either group.

**Figure 4:**
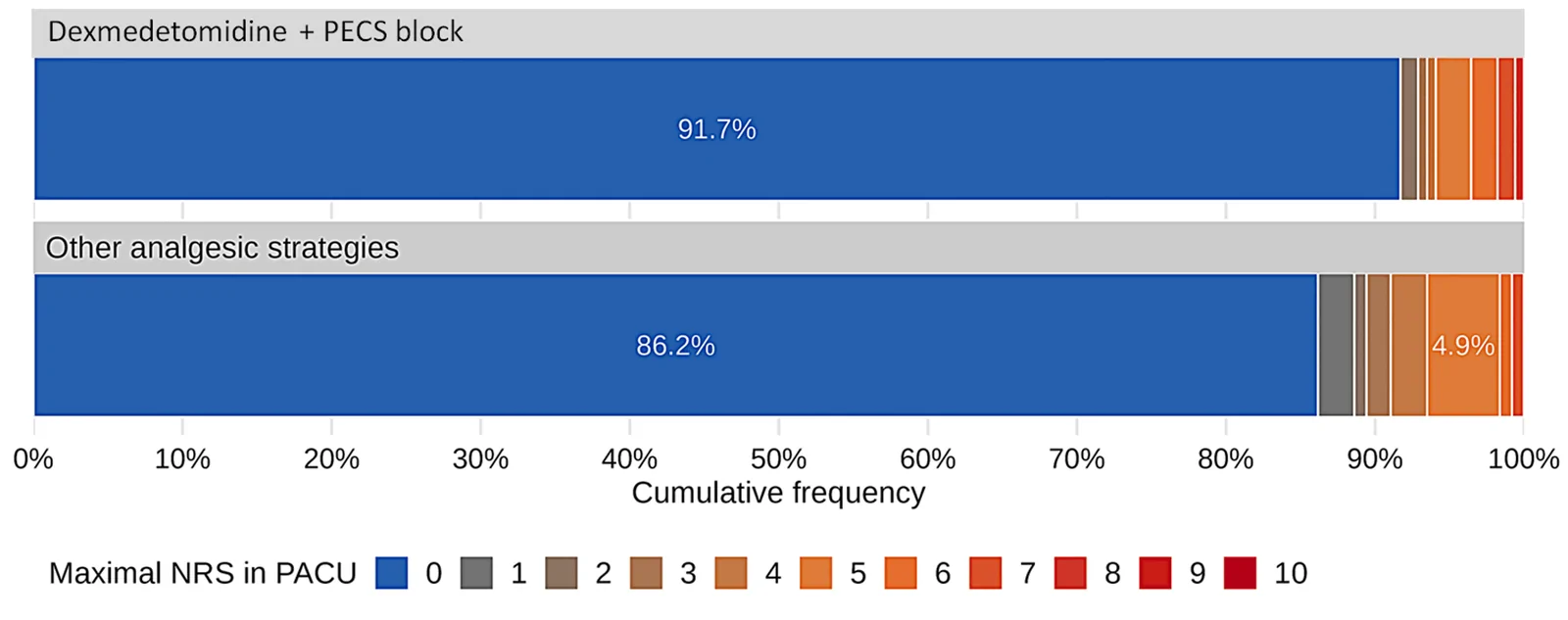
Maximum postoperative pain scores in the PACU (mastectomy + lumpectomy). Distribution of maximum numeric rating scale (NRS) pain scores recorded in the post-anesthesia care unit (PACU). Stacked bars represent the cumulative frequency distribution of maximum NRS scores within each treatment group. Abbreviation: PECS = pectoral nerve block.

**Figure 5:**
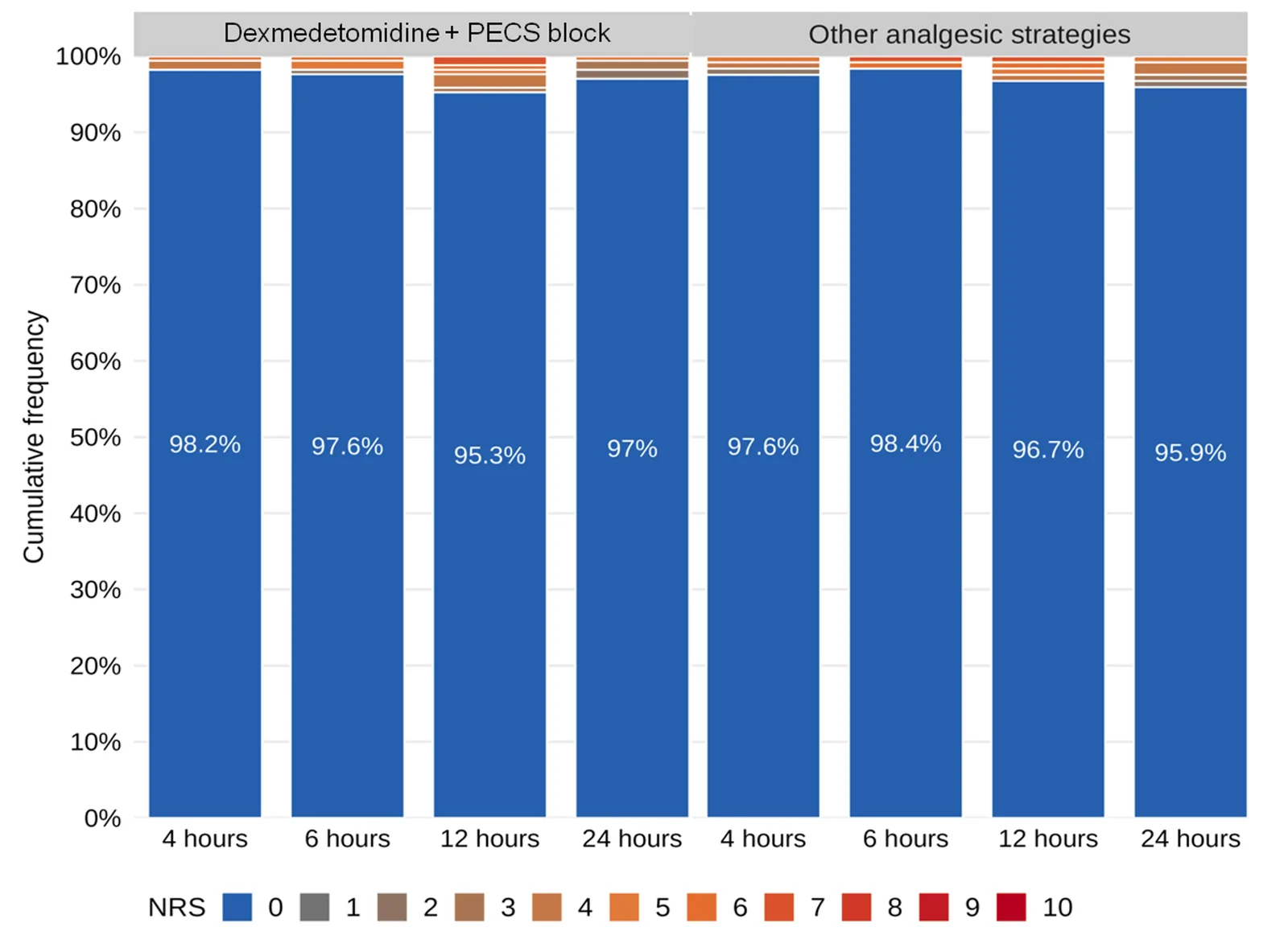
Postoperative pain scores during the first 24 hours after surgery (mastectomy and lumpectomy). Postoperative numeric rating scale (NRS) pain trajectories are shown across assessed time points. Stacked bars represent the cumulative frequency distribution of NRS scores at each postoperative assessment time point. Abbreviation: PECS = pectoral nerve block.

**Table 6:** Postoperative pain scores during the first 24 hours after surgery (mastectomy and lumpectomy). Continuous variables were analyzed using Welch’s t-test or the Brunner–Munzel test, as appropriate. P < 0.05 was considered statistically significant. Mean differences are reported as PECS block + dexmedetomidine minus other analgesic strategies. Brunner–Munzel values represent the odds of stochastic superiority (p̂/[1−p̂], where p̂ = P[Other > PECS+Dex] + 0.5·P[Other = PECS+Dex]). A value of 1 indicates no difference, values >1 indicate a stochastically larger value in the "Other analgesic strategies" group, and values <1 indicate a stochastically larger value in the "PECS block +Dexmedetomidine" group. Abbreviations: PECS = pectoral nerve block; NRS = numeric rating scale; PACU = post-anesthesia care unit; SD = standard deviation; Q1 = first quartile; Q3 = third quartile; CI = confidence interval; hrs = hours.

Characteristics		Pectoral block + dexmedetomidine	Other analgesic strategies	Eff.	95% CI	P
Max NRS in PACU	Mean (SD)	0.42 (1.5)	0.54 (1.5)	-0.12	-0.46; 0.23	0.511
	Median (Q1; Q3)	0 (0; 0)	0 (0; 0)	1.7	0.8; 3.6	0.177
NRS 4 hrs post surgery	Mean (SD)	0.08 (0.58)	0.09 (0.6)	-0.01	-0.15; 0.13	0.858
	Median (Q1; Q3)	0 (0; 0)	0 (0; 0)	1.4	0.27; 6.9	0.705
NRS 6 hrs post surgery	Mean (SD)	0.11 (0.72)	0.11 (0.83)	0	-0.18; 0.18	0.993
	Median (Q1; Q3)	0 (0; 0)	0 (0; 0)	0.69	0.12; 3.85	0.667
NRS 12 hrs post surgery	Mean (SD)	0.24 (1.1)	0.18 (1)	0.06	-0.19; 0.3	0.642
	Median (Q1; Q3)	0 (0; 0)	0 (0; 0)	0.68	0.2; 2.3	0.529
NRS 24 hrs post surgery	Mean (SD)	0.09 (0.54)	0.15 (0.74)	-0.06	-0.21; 0.1	0.467
	Median (Q1; Q3)	0 (0; 0)	0 (0; 0)	1.4	0.4; 5	0.606

The maximum pain scores recorded in the PACU were similarly low, with mean NRS values of 0.42 ± 1.50 in the PECS block plus dexmedetomidine group and 0.54 ± 1.50 in the comparator group (p = 0.511). Comparable findings were observed at all subsequent postoperative assessments, with no statistically significant differences between treatment strategies.

The uniformly low postoperative pain scores observed across the entire cohort are consistent with effective multimodal perioperative analgesia and resulted in only minimal discrimination between the evaluated analgesic strategies.

Rescue Analgesia

The proportion of patients requiring postoperative rescue analgesia (opioids + non-opioids) was comparable between treatment groups (Figure 6 and Table 7). Median time to first rescue analgesia was 6.2 hours in both groups, and Cox proportional hazards analysis demonstrated no statistically significant difference (HR: 0.92, 95% CI: 0.72-1.18; p = 0.495). Given that 85-90% of patients received rescue analgesia (predominantly non-opioid) within 24 hours and that scheduled and on-demand administrations could not be reliably distinguished in the electronic record, this near-identical median across groups is also consistent with a routine, protocol-driven dosing interval rather than exclusively pain-driven administration. This endpoint should be interpreted with this caveat.

**Figure 6:**
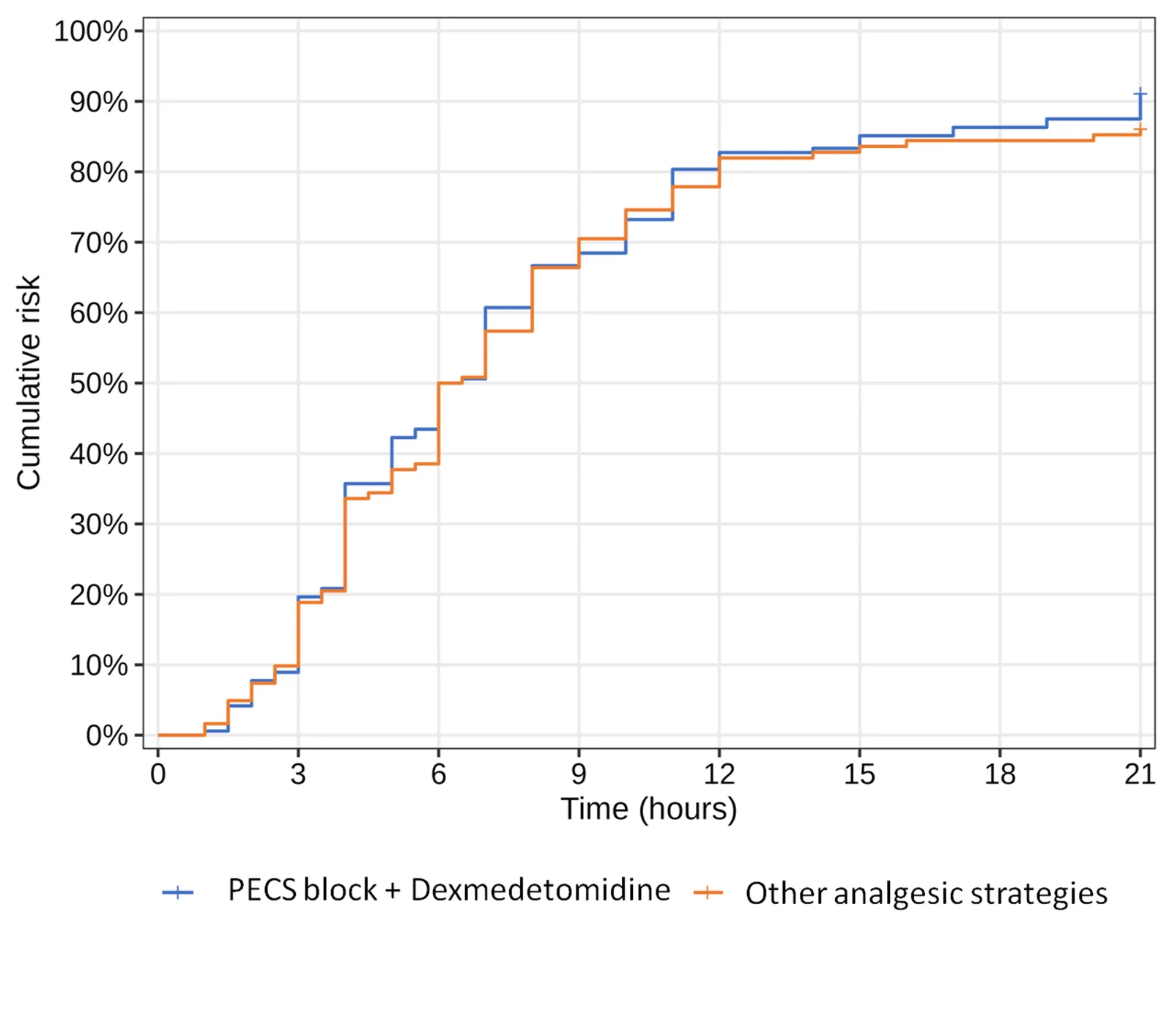
Time to first postoperative rescue analgesia (opioids + non-opioids in mastectomy + lumpectomy). Kaplan–Meier curves compare the combined pectoral nerve block plus dexmedetomidine strategy with other analgesic strategies. Abbreviations: PECS = pectoral nerve block.

**Table 7:** Time to first postoperative rescue analgesia, including opioids and non-opioids (mastectomy and lumpectomy). Time-to-event outcomes were evaluated using Kaplan–Meier analysis with log-rank testing and Cox proportional hazards regression. P < 0.05 was considered statistically significant. The reference group for the hazard ratio is "Other analgesic strategies"; HR < 1 favors the combined PECS + dexmedetomidine group. Abbreviations: PECS = pectoral nerve block; HR = hazard ratio; CI = confidence interval.

Time (hours)	PECS block + dexmedetomidine		Other analgesic strategies			
3	19.6 (13.4; 25.4)		18.9 (11.6; 25.5)			
6	50 (41.8; 57)		50 (40.3; 58.1)			
12	82.7 (76; 87.6)		82 (73.7; 87.6)			
20	87.5 (81.4; 91.6)		85.2 (77.4; 90.4)			
Median	6.2 (5.5; 7)		6.2 (6; 8)			
Comparison		HR		95% CI	P	
Other analgesic strategies		0.92		0.72; 1.18	0.495	

Although subgroup analyses suggested slight numerical differences between mastectomy and lumpectomy procedures during later postoperative follow-up, these exploratory findings were inconsistent across surgical subgroups and did not reach statistical significance. The overall and procedure-specific rescue analgesia findings are shown in Figures 6, 7 and Tables 7, 8.

**Figure 7:**
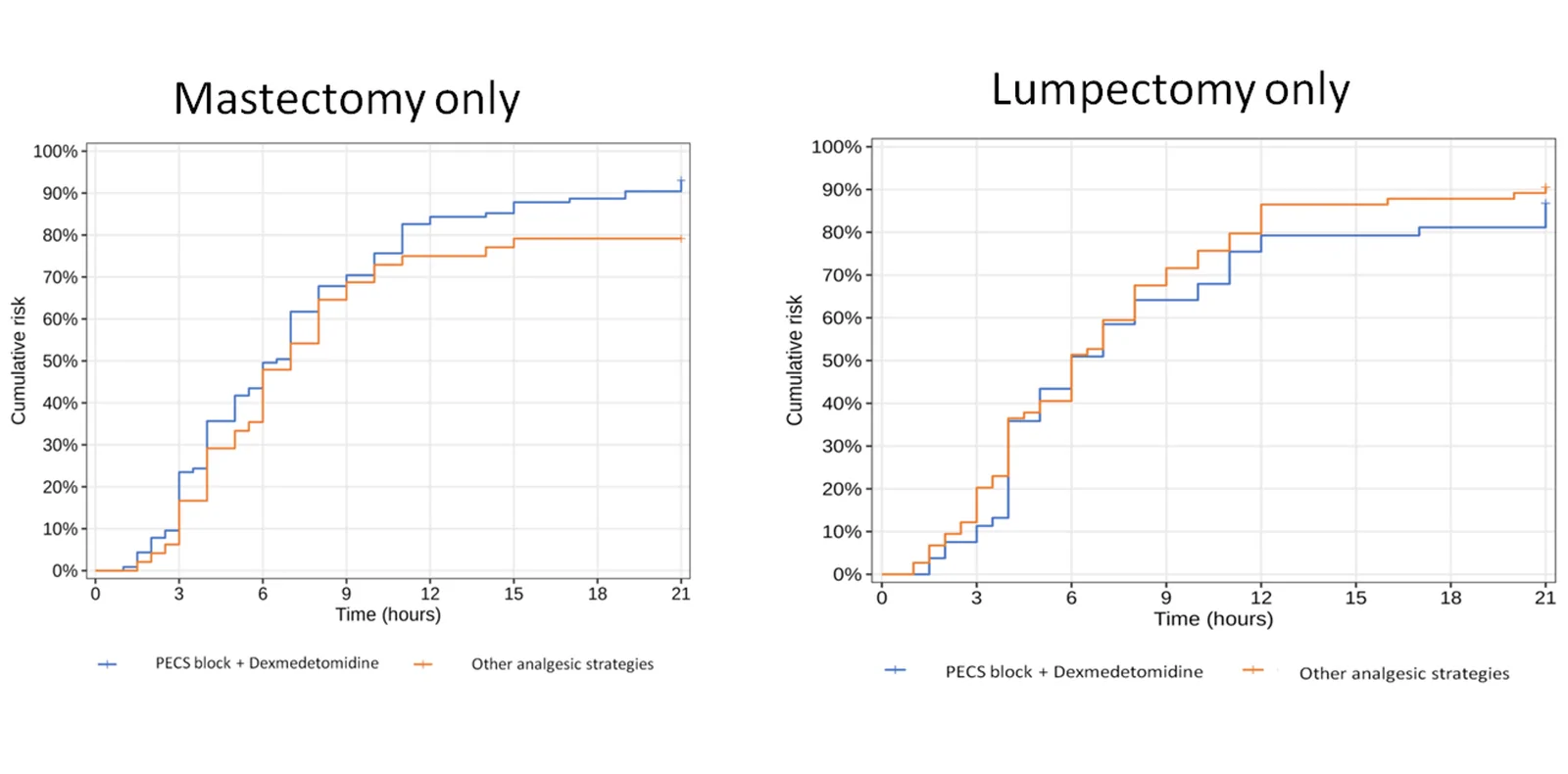
Procedure-specific time to first postoperative rescue analgesia, including opioids and non-opioids, stratified by procedure. Cumulative risk is shown over postoperative time for the combined pectoral nerve (PECS) block plus dexmedetomidine strategy and other analgesic strategies.

**Table 8:** Procedure-specific time to first postoperative rescue analgesia, including opioids and non-opioids, for mastectomy and lumpectomy. Time-point values are cumulative risk estimates with 95% confidence intervals. Time-to-event outcomes were evaluated using Kaplan–Meier analysis with log-rank testing and Cox proportional hazards regression. P < 0.05 was considered statistically significant. The reference group for the hazard ratio is "Other analgesic strategies"; HR < 1 favors the combined PECS + dexmedetomidine group. Abbreviations: PECS = pectoral nerve block; HR = hazard ratio; CI = confidence interval.

Mastectomy only						Lumpectomy only					
Time (hours)	Pectoral block + dexmedetomidine		Other analgesic strategies			Time (hours)	Pectoral block + dexmedetomidine		Other analgesic strategies		
3	23.5 (15.3; 30.8)		16.7 (5.4; 26.6)			3	11.3 (2.4; 19.5)		20.3 (10.6; 28.9)		
6	49.6 (39.5; 57.9)		47.9 (31.7; 60.3)			6	50.9 (35.5; 62.7)		51.4 (38.5; 61.5)		
12	84.3 (76.1; 89.8)		75 (59.2; 84.7)			12	79.2 (64.9; 87.7)		86.5 (76; 92.4)		
20	90.4 (83.2; 94.5)		79.2 (63.8; 88)			20	81.1 (67; 89.2)		89.2 (79.2; 94.4)		
Median	6.5 (5; 7)		7 (6; 9)			Median	6 (5; 10)		6 (5; 8)		
Comparison		HR		95% CI	P	Comparison		HR		95% CI	P
Other analgesic strategies		0.73		0.51; 1.06	0.103	Other analgesic strategies		1.16		0.80; 1.69	0.435

Analgesic consumption

While postoperative pain scores and rescue analgesia requirements were comparable between groups, clinically relevant differences emerged with regard to perioperative opioid exposure.

Nearly all patients received intraoperative non-opioid analgesics (166 (98.2%) versus 120 (97.6%)), and intraoperative opioids (168 (99.4%) versus 122 (99.2%)), reflecting the standardized institutional multimodal analgesic pathway. Postoperative analgesic requirements were remarkably low: 8 (4.8%) versus 10 (8.1%) patients required opioid administration in the PACU, while postoperative opioid administration on the surgical ward remained uncommon, 6 (3.6%) versus 3 (2.4%). During ward stay, 153 (90.5%) versus 105 (86.1%) patients received non-opioid analgesics, reflecting the institutional opioid-sparing protocol (Table 9). In Table 10, the procedure-specific perioperative analgesic use is presented.

**Table 9:** Perioperative analgesic use (mastectomy and lumpectomy). Categorical data are presented as n (%), with effect estimates and 95% confidence intervals, and were compared using Fisher’s exact test. P < 0.05 was considered statistically significant. Abbreviations: PECS = pectoral nerve block; PACU = post-anesthesia care unit; CI = confidence interval; n = sample size; PONV = postoperative nausea and vomiting.

Characteristics	PECS block + dexmedetomidine	Other analgesic strategies	Eff.	95% CI	P
Intraoperative non-opioid usage	166/169 (98.2%)	120/123 (97.6%)	1.01	0.97; 1.04	0.708
Intraoperative opioid usage	168/169 (99.4%)	122/123 (99.2%)	1.00	0.98; 1.02	0.842
Non-opioid usage in PACU	1/169 (0.6%)	2/123 (1.6%)	0.37	0.03; 3.99	0.459
Opioid usage in PACU	8/169 (4.8%)	10/123 (8.1%)	0.59	0.24; 1.44	0.253
Non-opioid usage in ward	153/169 (90.5%)	105/123 (86.1%)	1.05	0.96; 1.15	0.245
Opioid usage in ward	6/169 (3.6%)	3/123 (2.4%)	1.46	0.37; 5.71	0.618
PONV in PACU	1/169 (0.6%)	1/123 (0.8%)	-	-	1.00
PONV in ward	10/168 (6.0%)	6/123 (4.9%)	1.22	0.46; 3.25	0.80

**Table 10:** Intraoperative and postoperative opioid consumption expressed as morphine milligram equivalents (mastectomy and lumpectomy). Continuous variables were analyzed using Welch’s t-test or the Brunner–Munzel test, as appropriate. P < 0.05 was considered statistically significant. Mean differences are reported as PECS block + dexmedetomidine minus other analgesic strategies. Brunner–Munzel values represent the odds of stochastic superiority (p̂/[1−p̂], where p̂ = P[Other > PECS+Dex] + 0.5·P[Other = PECS+Dex]); a value of 1 indicates no difference, values >1 indicate a stochastically larger value in the "Other analgesic strategies" group, and values <1 indicate a stochastically larger value in the "PECS block + Dexmedetomidine" group. Abbreviations: PECS = pectoral nerve block; MME = morphine milligram equivalents; PACU = post-anesthesia care unit; SD = standard deviation; Q1 = first quartile; Q3 = third quartile; CI = confidence interval.

Characteristics (mastectomy + lumpectomy)		Pectoral block + dexmedetomidine	Other analgesic strategies	Eff.	95% CI	P
Intraoperative	Mean (SD)	17.7 (9.2)	50.7 (71.7)	-33	-46; -20.1	<0.001
	Median (Q1; Q3)	17.5 (15; 20.5)	16 (9.2; 64.8)	0.91	0.59; 1.41	0.733
PACU	Mean (SD)	0.31 (1.4)	0.5 (2)	-0.19	-0.61; 0.22	0.362
	Median (Q1; Q3)	0 (0; 0)	0 (0; 0)	1.55	0.61; 3.95	0.365
Ward	Mean (SD)	0.2 (1.2)	0.15 (1.1)	0.04	-0.21; 0.3	0.743
	Median (Q1; Q3)	0 (0; 0)	0 (0; 0)	0.81	0.19; 3.47	0.776
Intraoperative/PACU/Ward	Mean (SD)	18.2 (9.3)	51.3 (71.5)	-33.1	-46; -20.3	<0.001
	Median (Q1; Q3)	17.5 (15; 20.5)	17.5 (10; 64.8)	0.97	0.63; 1.5	0.914
PACU/Ward	Mean (SD)	0.5 (1.9)	0.65 (2.3)	-0.15	-0.64; 0.34	0.548
	Median (Q1; Q3)	0 (0; 0)	0 (0; 0)	1.38	0.62; 3.1	0.438

In the mastectomy subgroup, opioid administration in the PACU was significantly less frequent in the combined group than in patients receiving other strategies (4/116, 3.4% vs. 6/49, 12.2%; p = 0.048; Table 11). No correction for multiple comparisons was applied, consistent with the exploratory nature of subgroup analyses stated in Methods.

**Table 11:** Procedure-specific perioperative analgesic use for mastectomy and lumpectomy. Categorical data are presented as n (%) with effect estimates and 95% confidence intervals and are compared using Fisher’s exact test. P < 0.05 was considered statistically significant. Abbreviations: PECS = pectoral nerve block; PACU = post-anesthesia care unit; CI = confidence interval; n = sample size.

Mastectomy only						Lumpectomy only					
Characteristics	Pectoral block + dexmedetomidine	Other analgesic strategies	Eff.	95% CI	P	Characteristics	Pectoral block + dexmedetomidine	Other analgesic strategies	Eff.	95% CI	P
Intraoperative non-opioid usage	114/116 (98.3%)	47/49 (95.9%)	1.02	0.96; 1.09	0.421	Intraoperative non-opioid usage	52/53 (98.1%)	73/74 (98.6%)	0.99	0.95; 1.04	0.835
Intraoperative opioid usage	115/116 (99.1%)	48/49 (98%)	1.01	0.97; 1.06	0.594	Intraoperative opioid usage	53/53 (100%)	74/74 (100%)	–	–	>0.999
Non-opioid usage in PACU	1/116 (0.9%)	1/49 (2%)	0.43	0.03; 6.68	0.598	Non-opioid usage in PACU	0/53 (0%)	1/74 (1.4%)	–	–	0.583
Opioid usage in PACU	4/116 (3.4%)	6/49 (12.2%)	0.28	0.08; 0.95	0.048	Opioid usage in PACU	4/53 (7.7%)	4/74 (5.4%)	1.42	0.37; 5.43	0.621
Non-opioid usage in ward	107/116 (92.2%)	38/49 (79.2%)	1.17	1.00; 1.36	0.026	Non-opioid usage in ward	46/53 (86.8%)	67/74 (90.5%)	0.96	0.84; 1.09	0.520
Opioid usage in ward	4/116 (3.4%)	1/49 (2%)	1.69	0.19; 14.7	0.701	Opioids usage in ward	2/53 (3.8%)	2/74 (2.7%)	1.40	0.20; 9.60	0.752

Mean intraoperative opioid consumption was 17.7 ± 9.2 MME in patients receiving combined PECS block and intravenous dexmedetomidine compared with 50.7 ± 71.7 MME in patients receiving alternative analgesic strategies, corresponding to a mean difference of −33.0 MME (95% CI: −46.0 to −20.1; p < 0.001); the corresponding non-parametric (Brunner-Munzel) comparison of medians (17.5 vs. 16.0 MME) was not significant (p = 0.733), reflecting a heavily right-skewed distribution in the comparator group (SD: 71.7, exceeding its own mean).

Intraoperative remifentanil use differed dramatically by analgesic strategy (0% dexmedetomidine alone, 0.6% combined PECS + dexmedetomidine, 34.6% PECS block alone, 77.8% neither intervention), and remifentanil-exposed patients had markedly higher MME (group means: 115-161) than remifentanil-free patients (group means: 6-18) within every stratum. A multivariable linear regression adjusting for surgical procedure confirmed the unadjusted mean-based difference (−35.6 MME, 95% CI: −47.0 to −24.2, p < 0.001). Once remifentanil-exposed patients were excluded, intraoperative opioid consumption was low and broadly comparable across all four analgesic strategies, and a two-group, procedure-adjusted regression restricted to this subset nominally favored the pooled comparator strategies (+4.6 MME, 95% CI: 2.9-6.4, p < 0.001).

Postoperative opioid consumption remained low in both groups regardless of analgesic strategy. Median opioid consumption in the PACU and in the surgical ward was 0 MME throughout the observation period, and neither postoperative nor cumulative opioid requirements differed significantly between treatment strategies (Table 11). Procedure-specific opioid consumption expressed as MME is presented in Table 12.

**Table 12:** Procedure-specific opioid consumption expressed as morphine milligram equivalents for mastectomy and lumpectomy. Continuous data are presented as mean (standard deviation) and median (first quartile; third quartile), with effect estimates and 95% confidence intervals. Continuous variables were analyzed using Welch’s t-test or the Brunner–Munzel test, as appropriate. P < 0.05 was considered statistically significant. Mean differences are reported as PECS block + dexmedetomidine minus other analgesic strategies. Brunner–Munzel values represent the odds of stochastic superiority (p̂/[1−p̂], where p̂ = P[Other > PECS+Dex] + 0.5·P[Other = PECS+Dex]); a value of 1 indicates no difference, values >1 indicate a stochastically larger value in the "Other analgesic strategies" group, and values <1 indicate a stochastically larger value in the "PECS block + dexmedetomidine" group. Abbreviations: PECS = pectoral nerve block; MME = morphine milligram equivalents; PACU = post-anesthesia care unit; SD = standard deviation; Q1 = first quartile; Q3 = third quartile; CI = confidence interval.

Mastectomy only							Lumpectomy only						
Characteristics		Pectoral block + dexmedetomidine	Other analgesic strategies	Eff.	95% CI	P	Characteristics		Pectoral block + dexmedetomidine	Other analgesic strategies	Eff.	95% CI	P
Intraoperative	Mean (SD)	18 (10.6)	62.3 (85.3)	-44.3	-68.9; -19.8	<0.001	Intraoperative	Mean (SD)	17.1 (4.9)	43 (60.4)	-25.88	-39.9; -11.8	<0.001
	Median (Q1; Q3)	17.5 (15; 20.5)	15 (5; 127.5)	0.81	0.4; 1.64	0.712		Median (Q1; Q3)	17.5 (15; 19)	17 (12; 25)	1	0.54; 1.84	0.996
Post-anesthesia care unit (PACU)	Mean (SD)	0.18 (0.99)	0.84 (2.7)	-0.66	-1.46; 0.14	0.106	Post-anesthesia care unit (PACU)	Mean (SD)	0.59 (2)	0.28 (1.4)	0.32	-0.3; 0.94	0.313
	Median (Q1; Q3)	0 (0; 0)	0 (0; 0)	3.92	1.06; 14.57	0.085		Median (Q1; Q3)	0 (0; 0)	0 (0; 0)	0.53	0.14; 2.08	0.387
Ward	Mean (SD)	0.15 (0.91)	0.08 (0.54)	0.07	-0.15; 0.29	0.539	Ward	Mean (SD)	0.3 (1.6)	0.2 (1.3)	0.1	-0.43; 0.62	0.709
	Median (Q1; Q3)	0 (0; 0)	0 (0; 0)	0.77	0.08; 7.56	0.811		Median (Q1; Q3)	0 (0; 0)	0 (0; 0)	0.7	0.1; 5.15	0.737
Intraoperative/PACU/Ward	Mean (SD)	18.3 (10.6)	63.2 (85)	-44.9	-69.4; -20.4	<0.001	Intraoperative/PACU/Ward	Mean (SD)	18 (5.5)	43.5 (60.3)	-25.5	-39.5; -11.4	<0.001
	Median (Q1; Q3)	17.5 (15; 20.5)	16 (8; 128)	0.92	0.46; 1.86	0.884		Median (Q1; Q3)	17.5 (15; 20.5)	17.5 (12; 25)	0.99	0.54; 1.83	0.986
PACU/Ward	Mean (SD)	0.32 (1.5)	0.91 (2.8)	-0.59	-1.43; 0.25	0.165	PACU/Ward	Mean (SD)	0.9 (2.4)	0.48 (1.8)	0.42	-0.38; 1.21	0.300
	Median (Q1; Q3)	0 (0; 0)	0 (0; 0)	3	0.95; 9.42	0.103		Median (Q1; Q3)	0 (0; 0)	0 (0; 0)	0.56	0.18; 1.78	0.347

Among patients receiving dexmedetomidine, administered doses were comparable irrespective of whether a PECS block had also been performed in the pooled cohort (33.0 ± 10.7 µg combined vs. 36.5 ± 14.9 µg dexmedetomidine alone; not statistically significant), with similar findings after normalization for body weight (Table 13 and Figure 8). This was not the case in the mastectomy subgroup, where dexmedetomidine dose differed significantly between the combined group and the dexmedetomidine-alone group (32.5 vs. 39.9 µg; p = 0.006; median p = 0.004); this procedure-specific dosing difference is examined further in the Discussion. No predefined hemodynamic thresholds governed initiation of a continuous infusion. Table 13 reports the cumulative dexmedetomidine dose administered across the entire case. Loading and maintenance components could not be separated, as this was not consistently distinguished in the anesthesia record and practice varied by individual clinician.

**Table 13:** Dexmedetomidine administration in patients receiving dexmedetomidine, shown for the combined cohort and separately for mastectomy and lumpectomy. Doses are presented in micrograms (mcg) and normalized to body weight (mcg/kg). Continuous data are presented as mean (standard deviation) and median (first quartile; third quartile), with 95% confidence intervals, and were analyzed using Welch’s t-test or the Brunner–Munzel test, as appropriate. P < 0.05 was considered statistically significant. Abbreviations: PECS = pectoral nerve block; SD = standard deviation; Q1 = first quartile; Q3 = third quartile; CI = confidence interval; mcg = micrograms; kg = kilograms.

Mastectomy + lumpectomy							Mastectomy only							Lumpectomy only						
Characteristics		Pectoral block + dexmedetomidine	Other analgesic strategies	Diff.	95% CI	P	Characteristics		Pectoral block + dexmedetomidine	Other analgesic strategies	Diff.	95% CI	P	Characteristics		Pectoral block + dexmedetomidine	Other analgesic strategies	Diff.	95% CI	P
Dexmedetomidine (mcg)	Mean (SD)	33 (10.7)	36.5 (14.9)	-3.58	-7.98; 0.81	0.108	Dexmedetomidine (mcg)	Mean (SD)	32.5 (11)	39.9 (7.4)	-7.38	-12.4; -2.38	0.006	Dexmedetomidine (mcg)	Mean (SD)	34 (10.1)	35.6 (16.4)	-1.61	-7.42; 4.2	0.583
	Median (Q1; Q3)	32 (24.2; 40)	34.2 (28.8; 40)	-2.2	-5.2; 1.3	0.167		Median (Q1; Q3)	32 (24.1; 38.8)	41.1 (35.2; 44.9)	-9.14	-14.1; -2.1	0.004		Median (Q1; Q3)	34.3 (27.5; 40)	32 (28; 40)	2.03	-2.53; 8	0.578
Dexmedetomidine (mcg/kg)	Mean (SD)	0.484 (0.164)	0.501 (0.232)	-0.017	-0.085; 0.052	0.625	Dexmedetomidine (mcg/kg)	Mean (SD)	0.491 (0.174)	0.575 (0.167)	-0.084	-0.194; 0.026	0.123	Dexmedetomidine (mcg/kg)	Mean (SD)	0.469 (0.14)	0.479 (0.246)	-0.011	-0.097; 0.075	0.804
	Median (Q1; Q3)	0.474 (0.377; 0.576)	0.49 (0.352; 0.58)	-0.016	-0.053; 0.038	0.974		Median (Q1; Q3)	0.475 (0.381; 0.576)	0.615 (0.479; 0.654)	-0.14	-0.206; 0.013	0.087		Median (Q1; Q3)	0.474 (0.358; 0.542)	0.481 (0.35; 0.516)	-0.007	-0.069; 0.097	0.688

**Figure 8:**
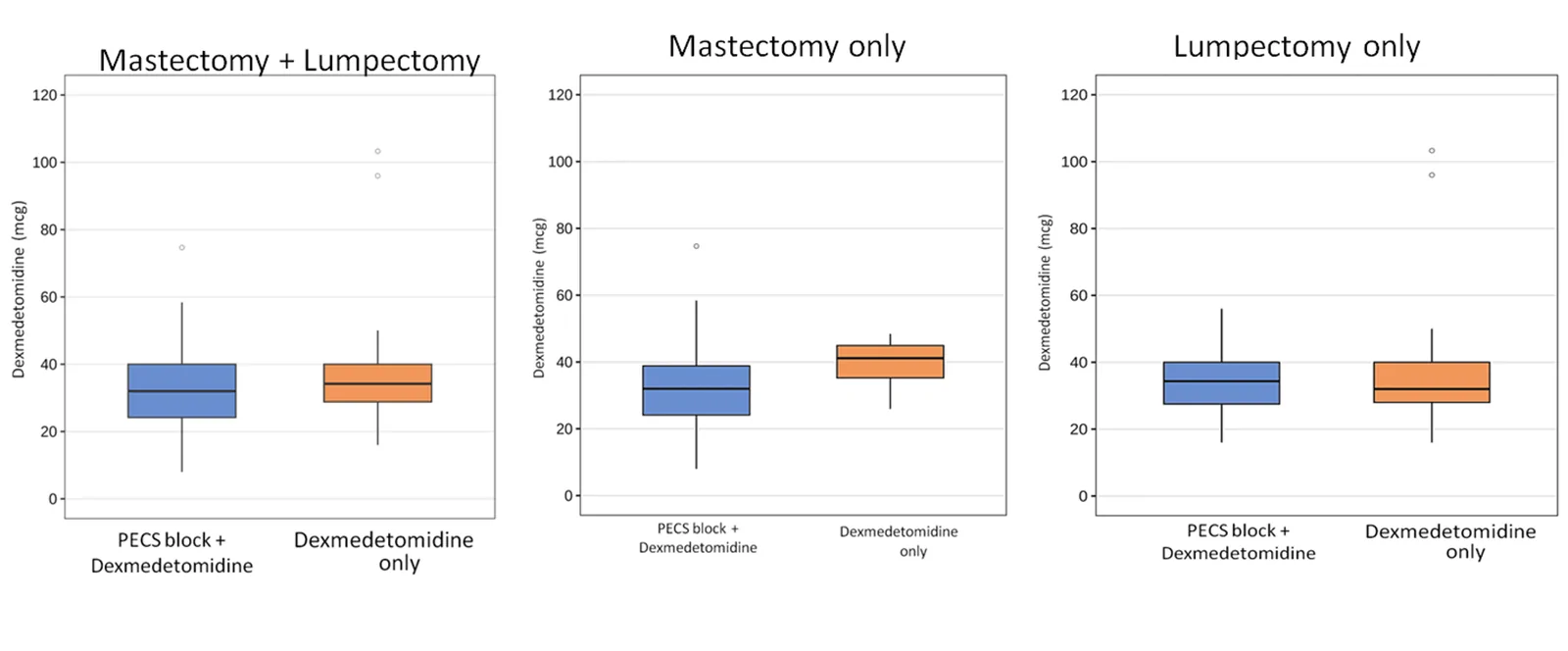
Dexmedetomidine dose distribution in patients receiving dexmedetomidine, shown for the combined cohort, mastectomy, and lumpectomy. Box plots compare patients receiving a pectoral nerve (PECS) block plus dexmedetomidine with those receiving dexmedetomidine only. Boxes represent the interquartile range, horizontal lines the median, whiskers the observed distribution, and individual points outlying values.

Taken together, postoperative analgesic outcomes remained uniformly favorable across all four analgesic strategies, while the difference in intraoperative opioid consumption between groups did not persist once anesthetic-technique confounding was accounted for. Perioperative analgesic use, opioid consumption, dexmedetomidine dosing, and the remifentanil-stratified sensitivity analysis are presented in Tables 9-14 and Figure 8.

**Table 14:** Sensitivity analyses of intraoperative opioid consumption accounting for anesthetic maintenance technique. Remifentanil use is strongly anti-correlated with dexmedetomidine use and associated with an approximately 10-fold higher intraoperative MME in every analgesic-strategy stratum. Once remifentanil-exposed cases are excluded, intraoperative opioid consumption is low and broadly comparable across all four strategies; the remifentanil-free "PECS alone" (n = 34) and "Neither" (n = 4) strata should be interpreted cautiously given limited sample size. Mean differences are reported as PECS block + dexmedetomidine minus other analgesic strategies. Brunner–Munzel values represent the odds of stochastic superiority (p̂/[1−p̂], where p̂ = P[Other > PECS+Dex] + 0.5·P[Other = PECS+Dex]); a value of 1 indicates no difference, values >1 indicate a stochastically larger value in the "Other analgesic strategies" group, and values <1 indicate a stochastically larger value in the "PECS block + Dexmedetomidine" group. Abbreviations: MME = morphine milligram equivalents; CI = confidence interval; PECS+Dex = PECS block + dexmedetomidine.

Analysis	Group	n	Mean MME	Median MME
Remifentanil-exposed patients	PECS+Dex	1	114.8	114.8
	PECS block alone	18	155.4	161.5
	Dexmedetomidine alone	0	—	—
	Neither	14	160.9	134.8
Remifentanil-free patients	PECS+Dex	168	17.1	17.5
	PECS block alone	34	5.8	5.0
	Dexmedetomidine alone	53	17.5	16.5
	Neither	4	14.8	14.5
Remifentanil use, % of group	PECS+Dex	169	0.6%	—
	PECS block alone	52	34.6%	—
	Dexmedetomidine alone	53	0.0%	—
	Neither	18	77.8%	—
Regression estimates	Comparison	Adjustment	Estimate (95% CI)	P
Multivariable linear regression	PECS+Dex vs. Other, all patients	Procedure	−35.6 MME (−47.0 to −24.2)	<0.001
Multivariable linear regression	PECS+Dex vs. Other, remifentanil-free (n = 259)	Procedure	+4.6 MME (2.9 to 6.4)	<0.001

Postoperative nausea and vomiting (PONV) was uncommon and did not differ significantly between groups, occurring in 1/169 (0.6%) versus 1/123 (0.8%) patients in the PACU (p = 1.00) and 10/168 (6.0%) versus 6/123 (4.9%) patients in the ward (p = 0.80), as seen in Table 9.

Physiological response

Intraoperative heart rate decreased in both treatment groups between induction of anesthesia and surgical readiness, indicating effective attenuation of the physiological response to surgical stimulation (Table 15). Patients receiving combined PECS block and intravenous dexmedetomidine had a slightly higher baseline heart rate at induction (85.6 ± 17.2 vs. 81.0 ± 14.0 beats/min; p = 0.014). However, heart rate immediately before surgical incision was comparable between groups (69.3 ± 13.2 vs. 70.8 ± 14.3 beats/min; p = 0.386).

**Table 15:** Intraoperative hemodynamics (mastectomy + lumpectomy). Continuous variables were analyzed using Welch’s t-test or the Brunner–Munzel test, as appropriate, and categorical variables were analyzed using Fisher’s exact test. P < 0.05 was considered statistically significant. Mean differences are reported as PECS block + dexmedetomidine minus other analgesic strategies. Brunner–Munzel values represent the odds of stochastic superiority (p̂/[1−p̂], where p̂ = P[Other > PECS+Dex] + 0.5·P[Other = PECS+Dex]); a value of 1 indicates no difference, values >1 indicate a stochastically larger value in the "Other analgesic strategies" group, and values <1 indicate a stochastically larger value in the "PECS block + dexmedetomidine" group. Abbreviations: PECS = pectoral nerve block; CI = confidence interval; SD = standard deviation; Q1 = first quartile; Q3 = third quartile; BPM = beats per minute; n = sample size.

Characteristics		Pectoral block + dexmedetomidine	Other analgesic strategies	Effect	95% CI	P
Atropine or glycopyrronium use		24/169 (14.3%)	23/123 (18.7%)	0.76	0.45; 1.29	0.318
Glycopyrronium		15/169 (8.9%)	11/123 (8.9%)	0.99	0.47; 2.09	0.978
Atropine		10/169 (6%)	12/123 (9.8%)	0.61	0.27; 1.37	0.238
Bradycardia		77/169 (45.6%)	43/123 (35%)	1.3	0.97; 1.75	0.07
Heart rate at induction (BPM)	Mean (SD)	85.6 (17.2)	81 (14)	4.5	0.927; 8.1	0.014
	Median (Q1; Q3)	84 (74; 95)	80 (73; 88)	4	-1; 9	0.016
Heart rate at ready (BPM)	Mean (SD)	69.3 (13.2)	70.8 (14.3)	-1.4	-4.7; 1.8	0.386
	Median (Q1; Q3)	67 (60; 75)	68 (60.5; 78)	-1	-5; 4	0.552
Heart rate change (%)	Mean (SD)	-17.1 (16.8)	-11.7 (16.3)	-5.4	-9.2; -1.5	0.007
	Median (Q1; Q3)	-18.6 (-28.2; -7.7)	-13.5 (-23.3; -4.1)	-5.1	-8.9; 0	0.01

Despite similar heart rates at surgical readiness, the relative reduction in heart rate from induction to incision was significantly greater in the combined PECS block and dexmedetomidine group. Mean heart rate decreased by 17.1 ± 16.8% compared with 11.7 ± 16.3% in patients receiving alternative analgesic strategies, corresponding to a mean difference of −5.4 percentage points (95% CI: −9.2 to −1.5; p = 0.007). Median relative heart rate reduction showed a similar pattern (−18.6% vs. −13.5%; p = 0.010).

Bradycardia was documented more frequently in the combined treatment group in 77 (45.6%) versus 43 (35.0%) patients, although this difference did not reach statistical significance (p = 0.070). Importantly, the increased incidence of recorded bradycardia was not accompanied by a higher requirement for pharmacological intervention. Atropine or glycopyrronium was administered to 24 (14.3%) versus 23 (18.7%) patients (p = 0.318), with glycopyrronium administered to 15 (8.9%) versus 11 (8.9%) patients and atropine to 10 (6.0%) versus 12 (9.8%) patients.

Exploratory analysis according to individual analgesic strategies showed that the attenuation of heart rate was associated with receipt of a PECS block (PECS + dexmedetomidine: −17.1%; PECS block alone: −16.4%; p = 0.804 between them) rather than with dexmedetomidine (dexmedetomidine alone: −8.0%, p < 0.001 vs. PECS + dexmedetomidine; neither intervention: −9.0%, p = 0.058 vs. PECS + dexmedetomidine; Table 15). Clinically relevant tachycardic responses were not observed in any treatment group. Intraoperative hemodynamic findings are summarized in Table 15.

Exploratory subgroup analyses

Predefined exploratory subgroup analyses were performed separately for patients undergoing mastectomy and lumpectomy to evaluate the consistency of the observed findings across surgical procedures.

Among patients undergoing mastectomy, postoperative analgesic outcomes remained comparable between treatment groups. Although patients receiving PECS block combined with intravenous dexmedetomidine demonstrated a numerically earlier median time to first rescue analgesic administration than those managed with alternative strategies (6.5 vs. 7.0 hours), the difference did not reach statistical significance (HR: 0.73, 95% CI: 0.51-1.06; p = 0.103).

Within the four-group exploratory analysis of mastectomy patients, the overall comparison was not statistically significant (global p = 0.085). Pairwise analyses suggested a longer time to first postoperative analgesic administration in patients receiving PECS block alone compared with the combined PECS plus dexmedetomidine strategy (HR: 0.63, 95% CI: 0.41-0.99; p = 0.044), whereas no differences were observed for dexmedetomidine alone or no regional intervention. Given the limited sample sizes of several comparator groups, these findings should be interpreted cautiously as hypothesis-generating.

Among patients undergoing lumpectomy, postoperative analgesic outcomes likewise remained comparable between analgesic strategies. Time-to-event analyses demonstrated no evidence of differences in the timing of rescue analgesia between groups (HR: 1.16, 95% CI: 0.80-1.69; p = 0.435), and the corresponding four-group comparison was similarly non-significant (global p = 0.750). Overall, subgroup analyses demonstrated patterns consistent with those observed in the primary cohort and did not identify a reproducible procedure-specific advantage of any individual analgesic strategy.

A full four-group comparison of the main outcomes (intraoperative opioid consumption, heart-rate change, maximum NRS, and ward rescue-opioid rate) is presented in Table 16, with the small size of the "neither intervention" group (n = 18) explicitly flagged as limiting precision for that stratum.

**Table 16:** Four-group comparison of main outcomes. Heart-rate change p-values compare each group to the PECS+Dex reference group. n = 18 in the "Neither" group; estimates for this stratum should be interpreted with caution given the small sample size. Abbreviations: PECS = pectoral nerve block; NRS = numeric rating scale; PACU = post-anesthesia care unit; MME = morphine milligram equivalents.

Outcome	PECS block + dexmedetomidine (n = 169)	PECS block alone (n = 52)	Dexmedetomidine alone (n = 53)	Neither (n = 18)
Intraoperative MME, mean	17.7	57.6	17.5	128.5
Intraoperative MME, median	17.5	5	16.5	125
Heart rate change, mean % (p vs. PECS+Dex)	−17.1 (ref.)	−16.4 (p = 0.804)	−8.0 (p < 0.001)	−9.0 (p = 0.058)
Max NRS in PACU, mean	0.42	0.4	0.74	0.33
Ward rescue opioid, n (%)	5 (3.0%)	2 (3.8%)	1 (1.9%)	0 (0.0%)
Bradycardia, n (%)	77 (45.6%)	24 (46.2%)	14 (26.4%)	5 (27.8%)

## Discussion

Principal findings

In this real-world retrospective cohort study of patients undergoing oncologic breast surgery within a standardized multimodal analgesic pathway, postoperative pain scores remained uniformly low regardless of the perioperative analgesic strategy employed, and postoperative opioid requirements and clinically relevant bradycardia requiring treatment remained comparable between groups. Conventional patient-reported pain outcomes demonstrated only minimal between-group differences, reflecting excellent postoperative analgesia across the entire study population. Two complementary outcome measures initially appeared to distinguish between strategies: intraoperative opioid consumption and heart-rate attenuation. Closer analysis attributed both to confounding rather than to the combined strategy itself: the opioid finding was attributable to near-complete collinearity between analgesic strategy and anesthetic maintenance technique, and the heart rate finding to the PECS block component rather than dexmedetomidine.

These findings suggest that, in contemporary multimodal analgesic pathways, conventional postoperative pain scores alone may be insufficient to discriminate between different opioid-sparing analgesic strategies. Instead, combining patient-reported outcomes with objective measures such as intraoperative opioid exposure, time to rescue opioid administration, and physiological response to surgical stimulation may provide a more comprehensive assessment of perioperative analgesic efficacy. Given that only eight patients across the cohort received a ward rescue opioid, the primary outcome is best characterized as inconclusive rather than as evidence of no effect.

As a large retrospective real-world cohort from a tertiary referral center in the Middle East, the present study extends the predominantly European, North American, and East Asian literature by demonstrating comparable physiological and analgesic patterns within a highly diverse routine clinical population.

Multimodal analgesia and the postoperative floor effect

The uniformly low postoperative pain scores observed in the present study likely reflect the effectiveness of the standardized multimodal analgesic pathway implemented at our institution rather than a lack of efficacy of the evaluated analgesic strategies. Median NRS pain scores remained zero throughout the first 24 postoperative hours; only a small proportion of patients required rescue opioids in the PACU or in the surgical ward, and non-opioid analgesics were used during ward stay in 153 (90.5%) versus 105 (86.1%) patients. Collectively, these findings indicate that postoperative analgesia was highly effective across the entire cohort.

Enhanced recovery pathways and contemporary multimodal analgesic concepts have substantially improved postoperative pain management following breast cancer surgery during the past decade. As a result, postoperative pain scores have progressively declined, while routine opioid requirements have become considerably lower than those reported in earlier clinical trials [9-11,29]. Under these circumstances, conventional patient-reported pain scores may become increasingly susceptible to floor effects, thereby limiting their ability to discriminate between different opioid-sparing analgesic strategies [12,13].

Our findings are consistent with this concept. Although postoperative pain scores remained virtually identical between treatment groups, two objective perioperative outcome measures, intraoperative opioid consumption and heart-rate response, initially appeared to differ between groups despite comparable postoperative NRS scores. As detailed below, both differences proved attributable to confounding rather than to the analgesic strategy itself.

These observations suggest that assessment of perioperative analgesic efficacy should extend beyond postoperative pain intensity alone. Time to first rescue opioid administration, perioperative opioid exposure, and physiological responses to surgical stimulation provide complementary information regarding analgesic performance that is not captured by pain scores alone. Rather than replacing patient-reported outcomes, these objective measures may improve the evaluation of multimodal opioid-sparing analgesic pathways, particularly when postoperative pain is already well controlled.

The present findings highlight an important methodological consideration for future perioperative analgesia research. In contemporary multimodal analgesic pathways, postoperative pain scores may cluster at very low levels, thereby limiting their ability to discriminate between different opioid-sparing interventions. Under these circumstances, complementary outcome measures, including intraoperative opioid exposure, time to rescue opioid administration, and physiological response to surgical stimulation, may provide additional information regarding analgesic performance while preserving the central importance of patient-reported pain outcomes.

Opioid-sparing effects of combined PECS block and intravenous dexmedetomidine

Although the combined PECS block and intravenous dexmedetomidine strategy was associated with lower mean intraoperative opioid consumption, this finding did not withstand closer scrutiny. Two features of the data limit a mechanistic interpretation. First, the comparator group's opioid distribution was heavily right-skewed (SD exceeding the mean), and the corresponding non-parametric comparison of medians was not significant. Second, and more importantly, intraoperative remifentanil use was found to be almost mutually exclusive with dexmedetomidine use in this cohort (0-0.6% among dexmedetomidine recipients vs. 34.6-77.8% among non-recipients), and remifentanil exposure - not analgesic strategy - was associated with an approximately 10-fold higher intraoperative MME in every stratum. Once remifentanil-exposed patients were excluded, intraoperative opioid consumption was low and broadly comparable across all four analgesic strategies. This apparent reversal in the remifentanil-free subset was driven substantially by the small PECS-alone (n = 34) and "neither" (n = 4) strata rather than a consistent pattern across all components, and should not be over-interpreted.

We interpret this pattern as reflecting near-complete confounding between analgesic strategy and anesthetic maintenance technique rather than an independent opioid-sparing mechanism of the combined strategy. This is compounded by a structural circularity inherent to the retrospective design: intraoperative opioid dose was titrated by the same attending anesthesiologist who selected the analgesic strategy and, in most cases, the anesthetic maintenance technique, so exposure and outcome arise substantially from a single clinical decision rather than from two independently randomized factors. Both PECS block [14,15] and dexmedetomidine [16-20] have, individually, been shown in randomized trials to reduce perioperative opioid requirements through complementary mechanisms, regional blockade of afferent nociceptive transmission for the former, and central sympatholytic and analgesic mechanisms for the latter [21-23,28], and nothing in the present data contradicts that established evidence base. What the present retrospective cohort cannot support is the stronger claim that the combination, as practiced in this real-world pathway, provided additional intraoperative opioid-sparing benefit beyond what anesthetic technique choice alone would predict.

Excessive perioperative opioid administration remains associated with opioid-related adverse effects, opioid-induced hyperalgesia, and delayed recovery, which continues to motivate interest in opioid-sparing anesthetic techniques more broadly [3,4,24,30].

Physiological response as a complementary measure of perioperative analgesic efficacy

An additional finding of the present study was a significantly greater attenuation of the heart-rate response to surgical stimulation in patients receiving combined PECS block and intravenous dexmedetomidine compared with other strategies. Because dexmedetomidine has a well-recognized direct sympatholytic, heart rate-lowering pharmacologic effect, this finding could plausibly reflect drug pharmacology rather than analgesic efficacy. To distinguish between these explanations, we examined heart-rate change across all four individual analgesic strategies. Attenuation was closely similar between the two block-containing groups regardless of dexmedetomidine (PECS + dexmedetomidine: −17.1%; PECS block alone: −16.4%; p = 0.804 between them), while both non-block groups showed markedly smaller attenuation regardless of dexmedetomidine (dexmedetomidine alone: −8.0%; neither intervention: −9.0%). This pattern indicates that heart-rate attenuation tracked with receipt of the PECS block rather than with dexmedetomidine, the opposite association from what a purely pharmacologic, sympatholysis-driven explanation would predict.

This block-associated attenuation is mechanistically plausible. Regional blockade of afferent nociceptive and sympathetic traffic from the surgical field is an established physiological pathway by which nerve blocks blunt the intraoperative stress response [14,15,23]. However, we note important caveats before drawing a causal conclusion. Baseline heart rate at induction was significantly higher in the combined group than in the other strategies (85.6 vs. 81.0 beats/min; p = 0.014), which arithmetically permits a larger relative reduction even in the absence of a true mechanistic difference. Heart rate is also a nonspecific physiological variable influenced by multiple factors beyond nociception. We therefore present this finding as a hemodynamic observation associated with the regional block component of the combined strategy, rather than as definitive evidence of superior nociceptive control.

Implications for future perioperative analgesia research

The present findings have implications beyond the evaluation of PECS block and intravenous dexmedetomidine alone. As multimodal perioperative analgesic pathways continue to evolve, postoperative pain following breast cancer surgery has become increasingly well controlled, with many patients reporting minimal pain and requiring little or no rescue opioid medication. Under these circumstances, postoperative pain intensity alone may no longer provide sufficient discriminatory power to detect clinically meaningful differences between contemporary opioid-sparing analgesic strategies.

Our results suggest that future perioperative analgesia studies may benefit from incorporating complementary outcome measures alongside conventional patient-reported pain scores. Objective perioperative variables, including intraoperative opioid exposure, time to first rescue opioid administration, and physiological responses to surgical stimulation, appear to capture different aspects of analgesic performance that may not be reflected by postoperative pain scores alone. Importantly, these complementary outcomes should be regarded as additions rather than replacements for patient-reported pain assessment.

This multidimensional approach may be particularly relevant for studies evaluating increasingly effective multimodal analgesic pathways, where improvements in postoperative pain scores become progressively more difficult to demonstrate because baseline analgesic quality is already high. Future prospective studies should therefore consider broader outcome frameworks that integrate patient-reported outcomes with objective perioperative measures to provide a more comprehensive assessment of analgesic efficacy.

Strengths and limitations

The present study has several strengths. First, it reflects routine clinical practice within a large tertiary referral center in the Middle East, providing real-world evidence from a contemporary and highly diverse breast cancer population treated within a standardized multimodal analgesic pathway. This setting extends the predominantly European, North American, and East Asian evidence base by providing data from a distinct and internationally diverse healthcare population.

A further strength is the comprehensive evaluation of perioperative analgesic efficacy using complementary outcome measures. Rather than relying exclusively on postoperative pain scores, the present study combined patient-reported outcomes with perioperative opioid exposure and physiological response to surgical stimulation, thereby providing a broader assessment of opioid-sparing analgesic strategies.

The transparency of the sensitivity analyses is a further strength. Rather than reporting a single mean-based comparison, the study identified and reported a substantial confounding structure (near-complete collinearity between analgesic strategy and anesthetic maintenance technique) that would otherwise have gone unrecognized, illustrating a methodological pitfall relevant to future observational analgesia research. The four-group design further allowed examination of the individual and potentially interactive contributions of the PECS block and dexmedetomidine, rather than treating the combined strategy as an unexamined single unit. Finally, a statistical analysis plan was filed with the institutional review board prior to data access, and no imputation was required for the primary or secondary outcomes within the analyzed cohort.

Several limitations should be acknowledged. The retrospective single-center design precludes causal inference and remains susceptible to residual confounding despite predefined subgroup and sensitivity analyses. Analgesic strategies were not randomly assigned but reflected routine clinical practice, and the four groups differed significantly in surgical procedure. Given the moderate sample size and unequal treatment group sizes, propensity score matching was not considered appropriate because it would have substantially reduced the effective sample size and statistical precision; we instead performed multivariable linear regression for the continuous intraoperative opioid outcome, which does not discard observations.

Intraoperative opioid dose was titrated by the same attending anesthesiologist who selected the analgesic strategy and, in most cases, the anesthetic maintenance technique; exposure and outcome therefore arise substantially from a single clinical decision, and the association between strategy and intraoperative opioid dose should not be read as independent evidence of reduced nociceptive input. Consistent with this, intraoperative remifentanil use was found to be almost perfectly collinear with not receiving dexmedetomidine (0-0.6% vs. 34.6-77.8%), and the reduction in intraoperative opioid consumption in the combined group did not persist once remifentanil-exposed patients were excluded. No validated conversion factor for continuous remifentanil infusion to MME exists in comparable literature; studies quantifying intraoperative remifentanil exposure typically report it in its native units (µg or µg/kg) rather than converting it to morphine equivalents, reflecting remifentanil's ultra-short, context-independent half-life, which limits its comparability to bolus-dosed opioids on a cumulative-equivalence basis. The University of California, San Francisco (UCSF) framework [28] applied in this study was, to our knowledge, the only readily available institutional reference offering a remifentanil conversion factor at all; we used it for consistency across all opioids while directly testing its practical impact via the remifentanil-stratified sensitivity analysis.

The proportion of patients receiving the combined strategy increased from 39.5% (2020) to 70.3% (2022) and remained 63.8-68.4% (2023-2024) over the study period (chi-square p < 0.001), introducing temporal confounding that the sparse-event primary Cox model (eight total ward-opioid events) could not accommodate as an additional covariate.

The interpretation of near-universal zero NRS scores as evidence of excellent analgesia cannot exclude two competing explanations: pain was assessed at rest only, and documentation completeness for scheduled assessments was not separately audited in this retrospective dataset. An isolated significant difference in PACU opioid use within the mastectomy subgroup did not survive correction for multiple comparisons and is presented as hypothesis-generating only.

No adjusted Cox model was fitted for the primary time-to-event outcome, given that only eight total events were observed across the primary comparison, which falls below the commonly used threshold of at least 10 events per model covariate.

Pre-inclusion screening counts, including the number of patients assessed for eligibility and excluded by reason, were not retained as a separate log during data collection and could not be reconstructed retrospectively.

Dexmedetomidine dosing was not fully standardized. No predefined hemodynamic thresholds governed the decision to initiate a continuous infusion beyond the loading dose, which introduces dosage heterogeneity within the dexmedetomidine-treated groups. Because only the cumulative dexmedetomidine dose per case was captured, the reported per-kilogram values cannot be directly compared against the stated loading-dose range, as loading and maintenance components were not consistently distinguished in the anesthesia record. Similarly, rescue opioids were administered without a mandated fixed NRS threshold, which introduces practice variability into the primary outcome's trigger criterion.

The distribution of local anesthetic volume between the two PECS-block fascial planes followed a routine practice (approximately 10 mL between the pectoralis major and minor, with the remainder between the pectoralis minor and serratus anterior) but was not individually recorded or analyzed per patient, precluding assessment of whether volume distribution influenced outcomes. Similarly, the number and experience of block operators, the exact administered local anesthetic mass in mg and mg/kg, and the timing of block placement relative to induction were not consistently logged in the anesthesia record; no cases of local anesthetic systemic toxicity were observed or documented during the study period.

Taken together, the present findings suggest that evaluation of contemporary opioid-sparing analgesic strategies should extend beyond postoperative pain scores alone. A multidimensional assessment integrating patient-reported outcomes, perioperative opioid exposure, and physiological response may provide a more comprehensive understanding of analgesic efficacy within modern multimodal perioperative care.

## Conclusions

Postoperative pain scores and opioid requirements were uniformly low regardless of analgesic strategy, and the primary time-to-rescue-opioid outcome was inconclusive. Both apparent secondary "positive" findings - the opioid-sparing effect and the heart-rate attenuation - were attributable to confounding (anesthetic maintenance technique and PECS block status, respectively) rather than to the combined strategy itself. These findings illustrate a floor effect in postoperative pain scores under contemporary multimodal pathways, and highlight anesthetic-technique confounding, calendar-time drift, and single-clinician decision-making as underappreciated sources of bias in retrospective analgesia research more broadly.
